# Simultaneous Single-Cell Genome and Transcriptome Sequencing of Termite Hindgut Protists Reveals Metabolic and Evolutionary Traits of Their Endosymbionts

**DOI:** 10.1128/msphere.00021-22

**Published:** 2022-02-02

**Authors:** Michael E. Stephens, Jacquelynn Benjamino, Joerg Graf, Daniel J. Gage

**Affiliations:** a Department of Molecular and Cell Biology, University of Connecticut, Storrs, Connecticut, USA; b Azenta Life Sciences, South Plainfield, New Jersey, USA; c Communication Partners Group, New York, New York, USA; Nanjing Normal University

**Keywords:** carbon metabolism, endosymbionts, genetic competence, metagenomics, protists, single-cell methods, symbiosis, termites

## Abstract

Some of the protist species which colonize the hindguts of wood-feeding *Reticulitermes* termites are associated with endosymbiotic bacteria belonging to the genus *Endomicrobium*. In this study, we focused on the endosymbionts of three protist species from Reticulitermes flavipes, as follows: Pyrsonympha vertens, Trichonympha agilis, and *Dinenympha* species II. Since these protist hosts represented members of different taxa which colonize separate niches within the hindguts of their termite hosts, we investigated if these differences translated to differential gene content and expression in their endosymbionts. Following assembly and comparative genome and transcriptome analyses, we discovered that these endosymbionts differed with respect to some possible niche-specific traits, such as carbon metabolism. Our analyses suggest that species-specific genes related to carbon metabolism were acquired by horizontal gene transfer (HGT) and may have come from taxa which are common in the termite hind gut. In addition, our analyses suggested that these endosymbionts contain and express genes related to natural transformation (competence) and recombination. Taken together, the presence of genes acquired by HGT and a putative competence pathway suggest that these endosymbionts are not cut off from gene flow and that competence may be a mechanism by which members of *Endomicrobium* can acquire new traits.

**IMPORTANCE** The composition and structure of wood, which contains cellulose, hemicellulose, and lignin, prevent most organisms from using this common food source. Termites are a rare exception among animals, and they rely on a complex microbiota housed in their hindguts to use wood as a source of food. The lower termite, Reticulitermes flavipes, houses a variety of protists and prokaryotes that are the key players in the disassembly of lignocellulose. Here, we describe the genomes and the gene expression profiles of five *Endomicrobium* endosymbionts living inside three different protist species from *R. flavipes*. Data from these genomes suggest that these *Endomicrobium* species have different mechanisms for using carbon. In addition, they harbor genes that may be used to import DNA from their environment. This process of DNA uptake may contribute to the high levels of horizontal gene transfer noted previously in *Endomicrobium* species.

## INTRODUCTION

Among the wood-feeding lower termites, symbiotic protists which reside in the hindgut are often colonized by endosymbionts (1–4). In *Reticulitermes* spp. termites, both Oxymonadida (order) and Parabasalia (class) protists associate with endosymbiotic bacteria belonging to the genus *Endomicrobium* (phylum *Elusimicrobia*, class *Endomicrobia*) (2, 5–7). Members of *Endomicrobium* have been shown to comprise a significant portion of the core bacterial community in wood-feeding termites, such as Reticulitermes flavipes (8, 9). These endosymbiotic lineages are thought to have initiated their associations with hindgut protists approximately 40 to 70 million years ago (10) and arose from free-living relatives during multiple independent acquisition events (11). Vertical passage from one protist cell to its progeny has resulted in cospeciation, as inferred from congruent rRNA phylogenies (7, 10, 12, 13).

In addition to colonizing the cytoplasm of certain hindgut protist species, *Endomicrobium* spp. are ectosymbionts of protists (14) and can be free living as well (11, 15–17). Because of their distribution across these separate niches, they provide an opportunity for studying bacterial genome evolution across different association lifestyles, namely, free living, endosymbiotic, and ectosymbiotic.

To determine the differences between two *Endomicrobium* species that are closely related but with distinct lifestyles, a previous study compared genomes of a free-living *Endomicrobium*, Endomicrobium proavitum strain Rsa215 (16), and “*Candidatus* Endomicrobium trichonymphae” strain Rs-D17 (3), an endosymbiont (3, 18). The findings suggested that the transition from the free-living state to an intracellular lifestyle involved genome reduction, similar to that of endosymbionts of sap-feeding insects and many obligate intracellular pathogens. However, the intracellular strain Rs-D17 also incorporated genes, possibly from other termite gut inhabitants, by horizontal gene transfer (HGT) (18). For example, the genome of “*Ca*. Endomicrobium trichonymphae” Rs-D17 appeared to have acquired several pathways, including those that encode sugar and amino acid transporters and genes involved in amino acids biosynthesis (18). These findings suggested that, unlike the endosymbionts of sap-feeding insects, *Endomicrobium* species may not be completely cut off from gene flow (18).

We expand upon these studies by presenting and comparing near-complete draft genome and transcriptome sequences of three *Endomicrobium* organisms, which were assembled from single protist cells of three different species that inhabit the hindgut of *R. flavipes*. One of these protist species, Pyrsonympha vertens, lives attached to the oxic gut wall (19, 20), while the other two, namely, Trichonympha agilis and *Dinenympha* species II, are found in the more anoxic hindgut lumen. In addition, *P. vertens* and *Dinenympha* species II are both oxymonads, while *T. agilis* is a parabasalid.

The analyses indicate that these *Endomicrobium* species have differences in their gene content and expression, which are related to carbon usage and metabolism. Also, as seen previously in “*Ca*. Endomicrobium trichonymphae” Rs-D17, they have likely acquired genes from putative donor taxa that are commonly associated with termites. In addition, we describe data suggesting that these *Endomicrobium* species have retained competence genes which may allow them to import exogenous DNA and that perhaps have contributed to HGT. The genes involved in this pathway are conserved across several *Endomicrobium* species and were expressed in the endosymbionts examined in this study.

## RESULTS

### Phylogeny of protist hosts.

**FIG 1:**
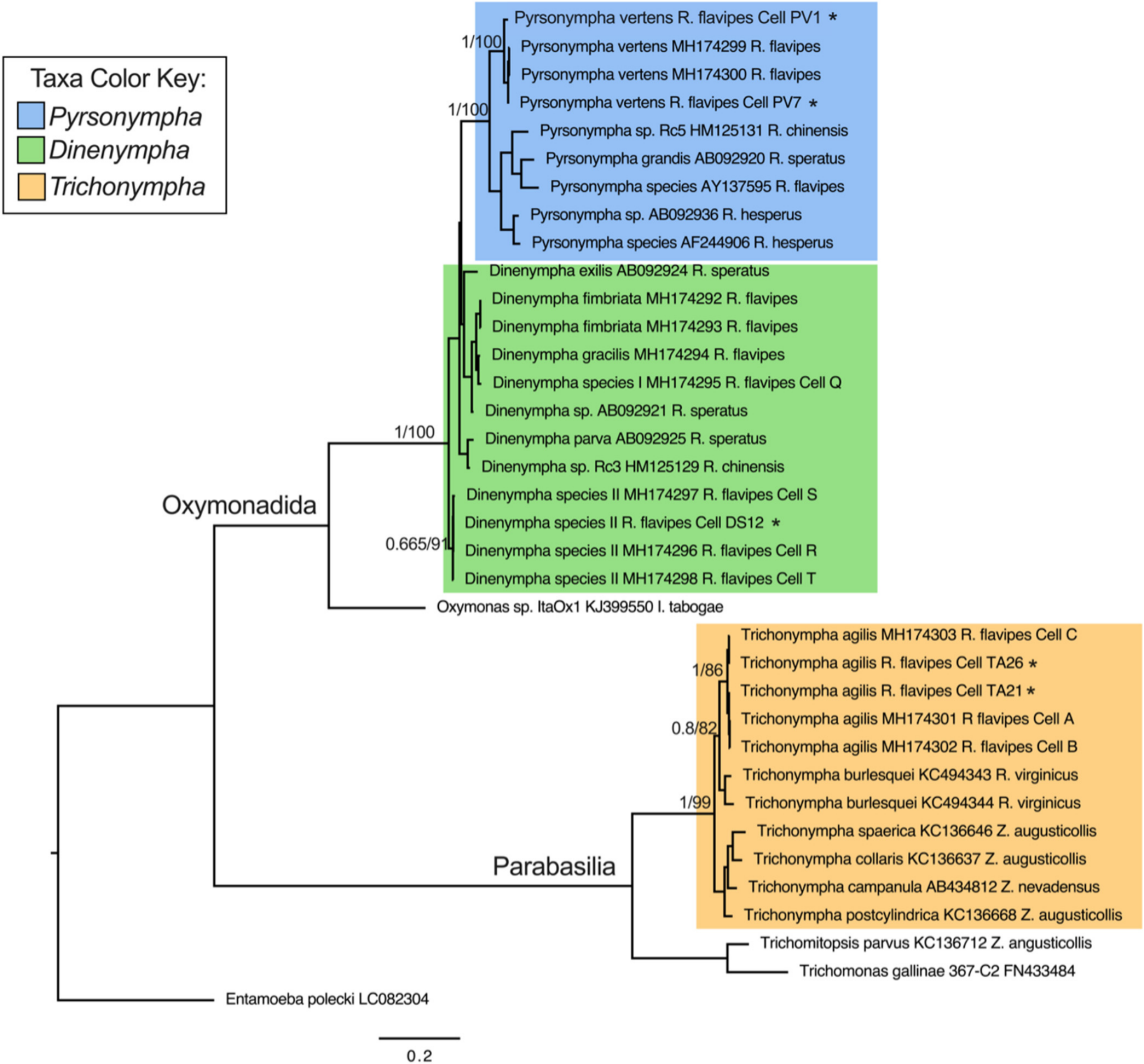
Protist 18S rRNA gene phylogeny. 18S rRNA genes were retrieved from single protist cell metagenome assemblies and aligned to references, and a maximum likelihood (ML) phylogenetic tree was made using IQ-TREE using substitution model TIM2+G4. All 18S rRNA gene sequences obtained in this study (denoted by *) are shown grouped with their respective references. Branch support values represent the Bayesian posterior probability and bootstrap support values, respectively.

Protist 18S rRNA genes were retrieved from metagenome assemblies and confirmed (when possible) independently by PCR and Sanger sequencing. A maximum likelihood (ML) phylogenetic tree was made that indicated that the species of the protist cells used in this study were *Trichonympha agilis* (cells TA21 and TA26), *Pyrsonympha vertens* (cells PV1 and PV7), and *Dinenympha* species II (cell DS12) (Fig. 1). These protist species have been confirmed previously to live associated with *R. flavipes*, the termite species used in this study.

### Taxonomic composition of the assemblies.

Reads from each of the single-cell metagenomes were mapped against a database of V4 amplicon sequence variants (ASVs) from previous work and from the DictDB and SILVA rRNA databases (Tab5 in Data Set S1 in the supplemental material). Each assembly, except PV7, contained reads that mapped only to the expected single-cell eukaryotic host. The PV7 metagenome contained no eukaryotic ASVs. The taxonomic composition of the PV1 and PV7 assemblies were the least diverse and contained the same major *Endomicrobium* ASV (Elusimicrobia_ASV024). This ASV was found associated with *Pyrsonympha* sp. previously (7). The PV7 assembly also contained single reads that matched Elusimicrobia_ASV020 and Elusimicrobia_ASV022. These ASVs are usually associated with *Trichonympha* sp. but may be associated with *Pyrsonympha* sp. at low levels or may be contaminants acquired during the single-cell isolation. The DS12 assembly was the most diverse and had reads mapping to a variety of spirochete species. This protist, *Dinenympha* sp. II, is known to associate with a large number of spirochete ectosymbionts (7). DS12 contained Elusimicrobia_ASV023 as the only *Endomicrobium* ASV. This ASV was seen previously to associate with *Dinenympha* sp. II (7). The *Trichonympha* assemblies TA21 and TA21 differed from each other in that reads from each hit different *Trichonympha* ASVs (18S_rRNA::TA21_scaffold229 and KC494354.1_Trichonympha, respectively) and different *Endomicrobium* ASVs (Elusimicrobia_ASV020 and Elusimicrobia_ASV019). It has been observed by us, and others, that there are at least two *Trichonympha* taxa in *R. flavipes* that are morphologically similar but that can be differentiated by their rRNA sequences and those of their resident *Endomicrobium* species (7, 21); TA21 and TA26 may be an example of each of these two taxa.

10.1128/msphere.00021-22.10 DATA SET S1 The spreadsheet contains the following tabs: Tab1, Amplicon metadata; Tab2, Filtering references; Tab3, Assembly information; Tab4, Genome references; Tab5, 16S-V4 ASV information; Tab6, HGT data; Tab7, RPKM values and gene information; Tab8, RPKM charts; Tab9, Biosynthetic genes; Tab10, Repair and competence genes; Tab11, Type IV/tad genes; Tab12, Pseudogenes. Download Data Set S1, XLSX file, 3.2 MB. Copyright © 2022 Stephens et al. 2022 Stephens et al. https://creativecommons.org/licenses/by/4.0/ This content is distributed under the terms of the Creative Commons Attribution 4.0 International license.

### *Endomicrobium* genome statistics.

**FIG 2:**
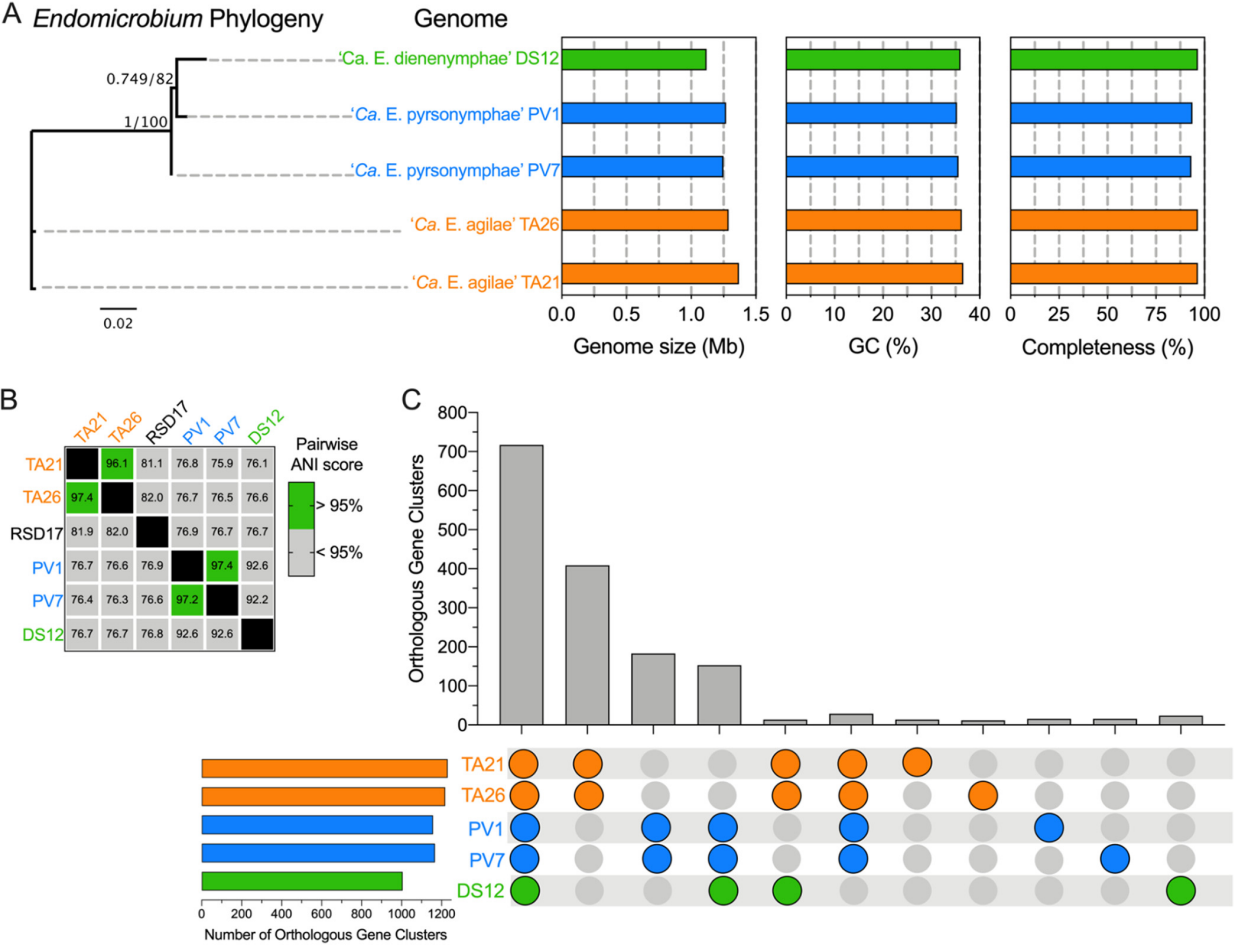
*Endomicrobium* draft genomes statistics, species identification, and shared gene content. (A) 16S rRNA gene maximum likelihood tree (unrooted) of the three *Endomicrobium* species, genome sizes, percent G+C content, and estimated percent genome completeness. (B) Pairwise genomic ANI scores of *Endomicrobium* genomes obtained by this study and a previously sequenced relative, Rs-D17. (C) UpSet graph of the number of orthologous gene clusters (OGCs) of protein-coding sequences within and across each of the *Endomicrobium* draft genomes.

Five near-complete *Endomicrobium* genomes were obtained from single protist cell metagenomic assemblies. The five draft genomes contained 25 to 229 contigs, ranged from 1.12 to 1.37 Mb long, and had a G+C content from 35.3% to 36.6%. NCBI annotated between 10 and 37 pseudogenes in the genomes. They are listed in Tab12 of Data Set S1. The genomes were 93.3% to 96.6% complete and contained between 0.0% and 5.2% contamination as measured by CheckM (Fig. 2A; also Tab3 in Data Set S1. Gene content, as determined by NCBI, is also provided in this table.

To determine if these genomes were from the same or different *Endomicrobium* species, we calculated pairwise genomic distances using average nucleotide identity (ANI) methods as implemented by JSpeciesWS (22). An ANI score of 95% or greater was used as a marker for species-level cutoff (Fig. 2B) (23). From *T. agilis* samples, we assembled two *Endomicrobium* draft genome sequence which had ANI scores of greater than 96% to one another but less than 90% to “*Ca*. Endomicrobium trichonymphae” Rs-D17, indicating that they are likely different species. Based on this analysis, we refer to the draft genomes as coming from “*Candidatus* Endomicrobium agilae” TA21 and “*Candidatus* Endomicrobium agilae” TA26. We also assembled two *Endomicrobium* genomes from *P. vertens* samples which had an ANI score greater than 97% identity to each another (Fig. 2B) and whose 16S rRNA genes were greater than 98% identical to a previously described species, “*Candidatus* Endomicrobium pyrsonymphae” (6), which is the *Candidatus* species designation that we use for PV1 and PV7. One additional *Endomicrobium* genome sequence was assembled from *Dinenympha* species II. This genome did not share an ANI score greater than 95% to other *Endomicrobium* genomes and was thus given a new *Candidatus* species designation, namely, “*Candidatus* Endomicrobium dinenymphae” DS12 (Fig. 2B).

Individually, these *Endomicrobium* genomes contained between 1,005 and 1,230 orthologous gene clusters (OGCs), of which 717 were found in all 5 genomes (Fig. 2C). Additionally, 409 OCGs were unique to “*Ca*. Endomicrobium agilae” TA21 and TA26, and another 183 OGCs were unique to “*Ca*. Endomicrobium pyrsonymphae” PV1 and PV7 (Fig. 2C). Although the genome of “*Ca*. Endomicrobium dinenymphae” DS12 had only 24 unique OGCs, it shared 153 with “*Ca*. Endomicrobium pyrsonymphae” PV1 and PV7 (Fig. 2C) which may reflect similar selective pressures for gene retention in their oxymonad hosts *Dinenympha* and *Pyrsonympha* sp., or their more recent shared history, compared with the *Endomicrobium* sp. (TA21 and TA26) that associated with the parabasalid *T. agilis*.

Analyses using HGTector indicated that the 5 *Endomicrobium* genomes contained between 72 (TA26) and 100 (PV1) genes that had likely been acquired by HGT. This result represented roughly 7% of the protein-coding genes of these genomes. A total of 35 core genes appeared to have been acquired by HGT in all 5 organisms (Tab6 in Data Set S1). For comparison, HGTector identified 75 genes acquired by HGT in *Endomicrobium* strain RsD17 representing 7% of its genome.

### Biosynthesis of amino acids, vitamins, and peptidoglycan.

The presence of genes for the various functions discussed below were either annotated by NCBI or detected by tblastn using the queries listed in Tab9 and Tab10 of Data Set S1. When genes were not detected in this manner, read mapping using Geneious and read mapping using MEGAN (24) were done as well to identify reads from genes that may not have assembled into contigs. In general, each of the five *Endomicrobium* genome sequences assembled in this study had similar gene contents for processes involved in the biosynthesis of amino acids (see Fig. S2A in the supplemental material), vitamins (Fig. S2B), glycolysis and the pentose phosphate pathway (Fig. S2C), and peptidoglycan (Fig. S2D). Each genome possessed complete pathways for alanine (from cysteine) aspartate, arginine, glutamine, glutamate, glycine (from imported serine), isoleucine, leucine, valine, lysine, tyrosine, phenylalanine, and tryptophan biosynthesis (Fig. S2A). Interestingly, the *Endomicrobium* symbionts of oxymonad protists (PV1, PV7 and DS12) lacked at least one gene in the biosynthesis pathway for histidine (*hisG*) (Fig. S2A). The histidine biosynthetic pathway was complete in the genomes of “*Ca.* Endomicrobium agilae” TA21 and TA26 (Fig. S2A). Conversely, it is likely that the *Endomicrobium* symbionts of oxymonad protists (PV1 and PV7) can make proline, while the symbionts represented by genomes TA21, TA26, DS12, and RsD17 cannot (Fig. S2A). The five genomes encoded incomplete pathways for the synthesis of cysteine and methionine. The three genomes isolated from oxymonad protists encoded a methionine transporter (MetT) and all contained a gene encoding a B12-dependent methionine synthase system comprised of MetH and an activation protein MetH2 (Fig. S2A). However, it is unclear how methionine can be synthesized or transported in TA21 and TA26. A similar situation exists in the *Endomicrobium* strain RsD17 (18). Also incomplete in all five genomes were pathways for the synthesis of serine and asparagine. Each genome encoded a serine transporter (SdaC) and a proline transporter (ProT), and PV1, PV7, and DS12 each encoded a glutamate transporter (GltP) (Fig. S2A).

10.1128/msphere.00021-22.5 FIG S2 Gene content in *Endomicrobium* genomes regarding metabolic functions and cell wall biosynthesis. (A) Gene content of amino acid biosynthesis pathways. (B) Gene content of vitamins and cofactor biosynthesis pathways. (C) ps, pseudogene. Genes involved in central metabolism. (D) Gene content of peptidoglycan biosynthesis. Note that the genes marked with a “*” or a “!” were not in the final assemblies, but their reads were detected by either MEGAN (!) or by read mapping onto the same gene from another closely related organisms (*) or both (!*) (see Fig. S3 as an example). Download FIG S2, JPG file, 1 MB. Copyright © 2022 Stephens et al. 2022 Stephens et al. https://creativecommons.org/licenses/by/4.0/ This content is distributed under the terms of the Creative Commons Attribution 4.0 International license.

The five *Endomicrobium* genomes also had similar gene contents for processes involved in the biosynthesis of vitamins and cofactors, with the pathways to pantothenate, coenzyme A (CoA), NAD, and NADP being complete and other pathways being incomplete (Fig. S2B). Interestingly, the biotin biosynthesis pathways in the five genomes are missing just a single gene (*bioW*) needed to convert pimelate to pimelate-CoA suggesting that pimelate-CoA may be synthesized by another enzyme or imported (Fig. S2B). Several genes in the thiamine biosynthesis pathway were also missing in each of these genomes (Fig. S2B). As noted previously for *E. proavitum* and “*Candidatus* Endomicrobium trichonymphae” strain Rs-D17, the five genomes described here were also missing the steps in the folate pathway needed to make 4-aminobenzoate, which may be transported into the cells (18). The pathways for pyridoxine (B6) and vitamin B12 were also incomplete, although each of the five *Endomicrobium* genomes appeared to encode ABC transport systems for vitamin B12 and heme.

Regarding peptidoglycan synthesis, each *Endomicrobium* genome was missing a gene encoding the enzyme (BacA) which typically dephosphorylates undecaprenyl pyrophosphate (Fig. S2D). Since these different *Endomicrobium* species, including the free-living *E. proavitum*, are missing the same gene, it may be that these bacteria utilize an alternate phosphatase to carry out the same function as BacA.

### Differences in carbon metabolism.

Some of the more interesting differences between these *Endomicrobium* genomes pertained to carbon metabolism. Each of the five *Endomicrobium* genomes encoded relatively simple pathways for importing and using different wood-derived carbon sources. Each had a complete phosphotransferase system (PTS) for importing sugars. Present were two EIIA genes encoding sugar specific phosphorylation proteins related most closely to those of the mannose and fructose type EIIA proteins (Fig. S2C). Zheng et al. reported that *E. proavitum*, which contains a very similar PTS pathway, did not grow on mannose or fructose but did grow on glucose, suggesting that glucose may be the carbohydrate transported by the PTS in that *Endomicrobium* species and perhaps in the ones described here as well (16, 18).

**FIG 3:**
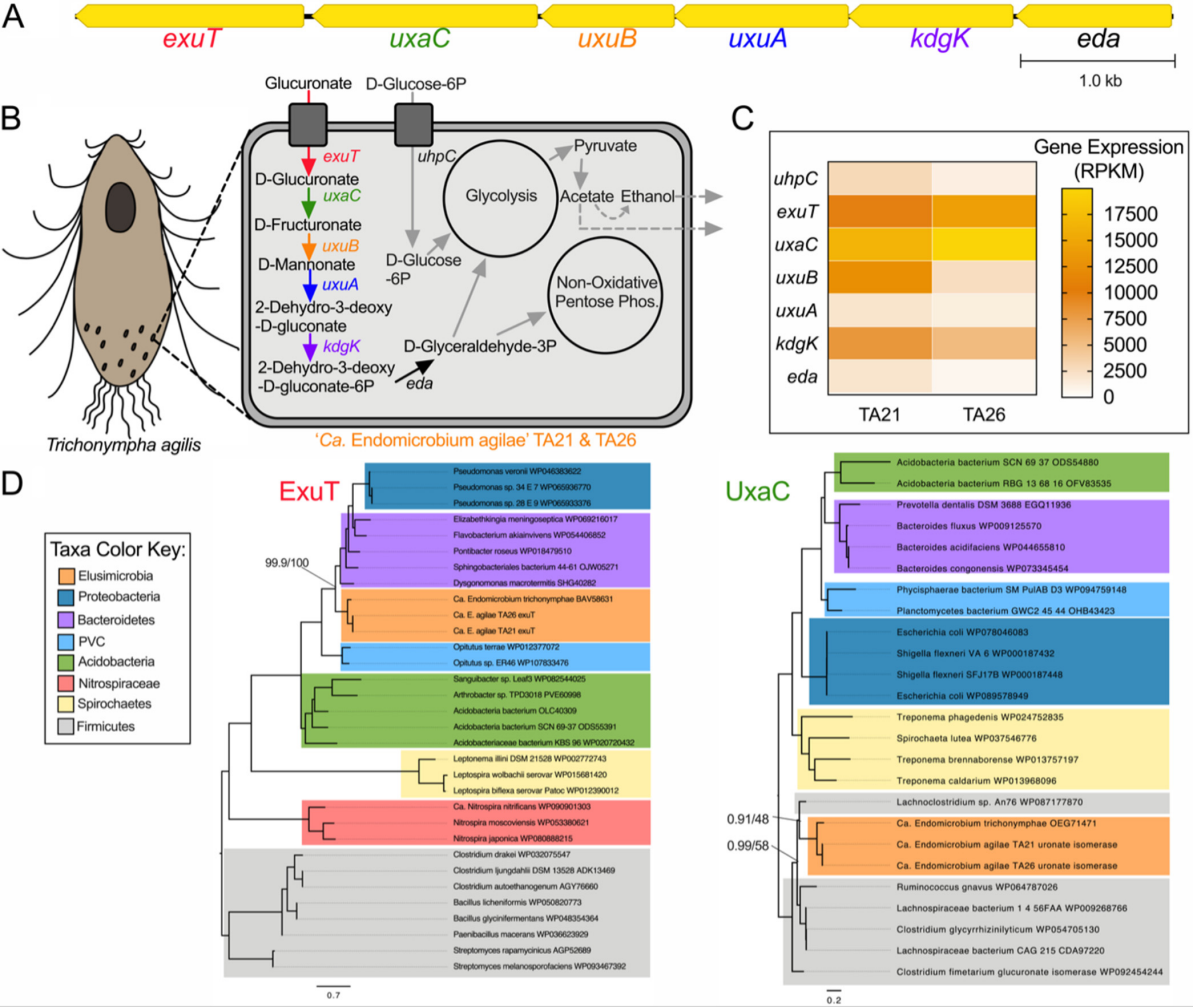
Carbon metabolism and HGT in “*Ca*. Endomicrobium agilae.” (A) Gene neighborhood of the genes involved in the metabolism of glucuronate in the “*Ca*. Endomicrobium agilae” TA21 and TA26 genomes. (B) Diagram of a protist host and an *Endomicrobium* cell showing the inferred metabolic conversions of carbon sources based on gene content data. (C) Gene expression data of genes of interest (rows) pertaining to carbon metabolism in “*Ca*. Endomicrobium agilae” TA21 and TA26 (columns). (D) Maximum likelihood phylogenetic trees of amino acid sequences of the transporter (ExuT, using substitution model LG+F+G4) and isomerase (UxaC, using substitution model LG+I+G4) in the glucuronate metabolism pathway. Support values represent the Bayesian posterior probability and bootstrap support values, respectively.

**FIG 4:**
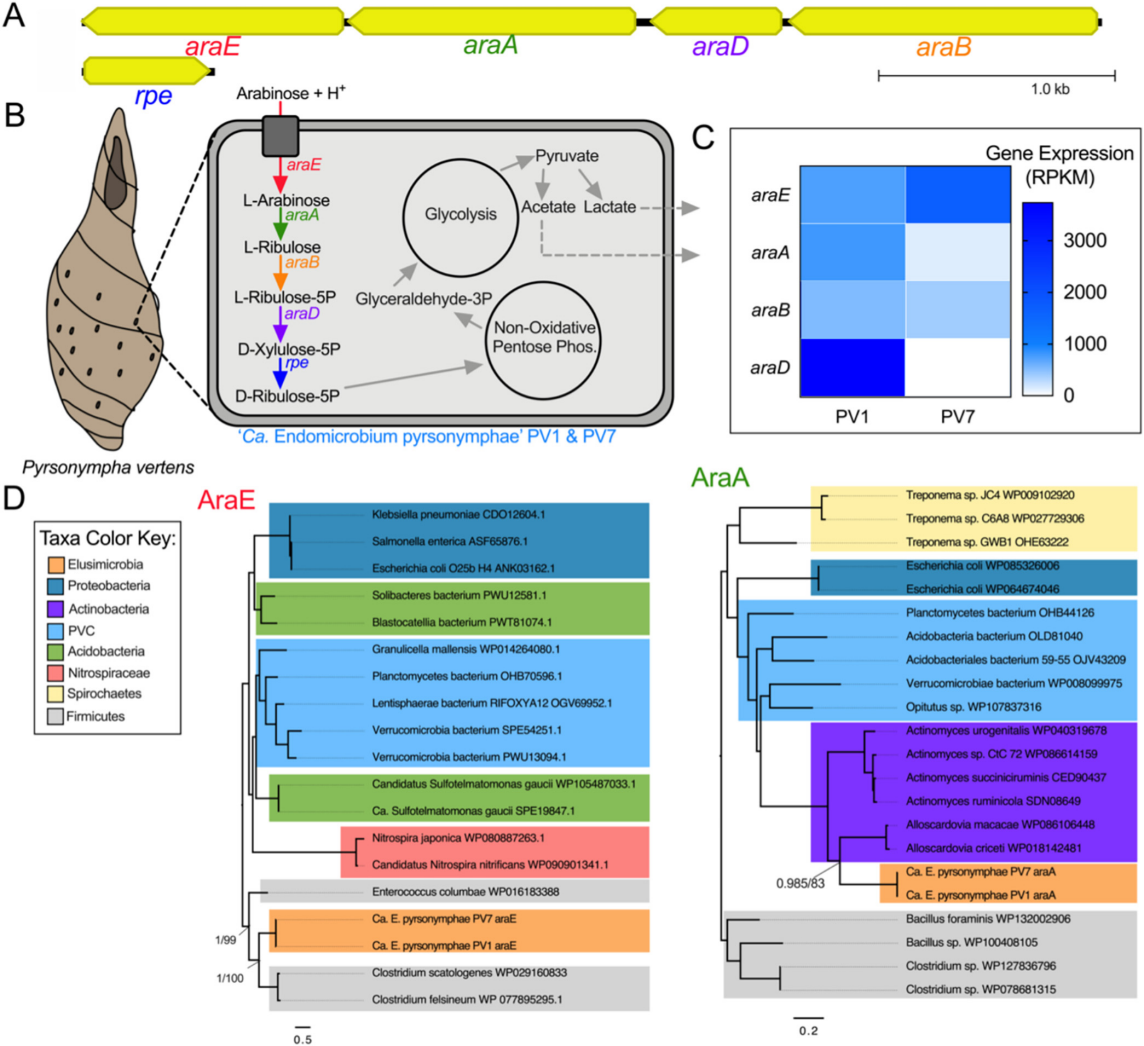
Carbon metabolism and HGT in “*Ca*. Endomicrobium pyrsonymphae.” (A) Gene neighborhood of the genes involved in the metabolism of arabinose in the “*Ca*. Endomicrobium pyrsonymphae” PV1 and PV7 genomes. (B) Diagram of a protist host and an *Endomicrobium* cell showing the inferred metabolic conversions of carbon sources based on gene content data. (C) Gene expression data of genes of interest (rows) pertaining to carbon metabolism in “*Ca*. Endomicrobium pyrsonymphae” PV1 and PV7 (columns). (D) Maximum likelihood phylogenetic trees of amino acid sequences from the transporter (AraE, using substitution model LG+F+G4) and isomerase (AraA, using substitution model LG+I+G4) in the arabinose metabolism pathway. Support values represent the Bayesian posterior probability and bootstrap support values, respectively.

**FIG 5:**
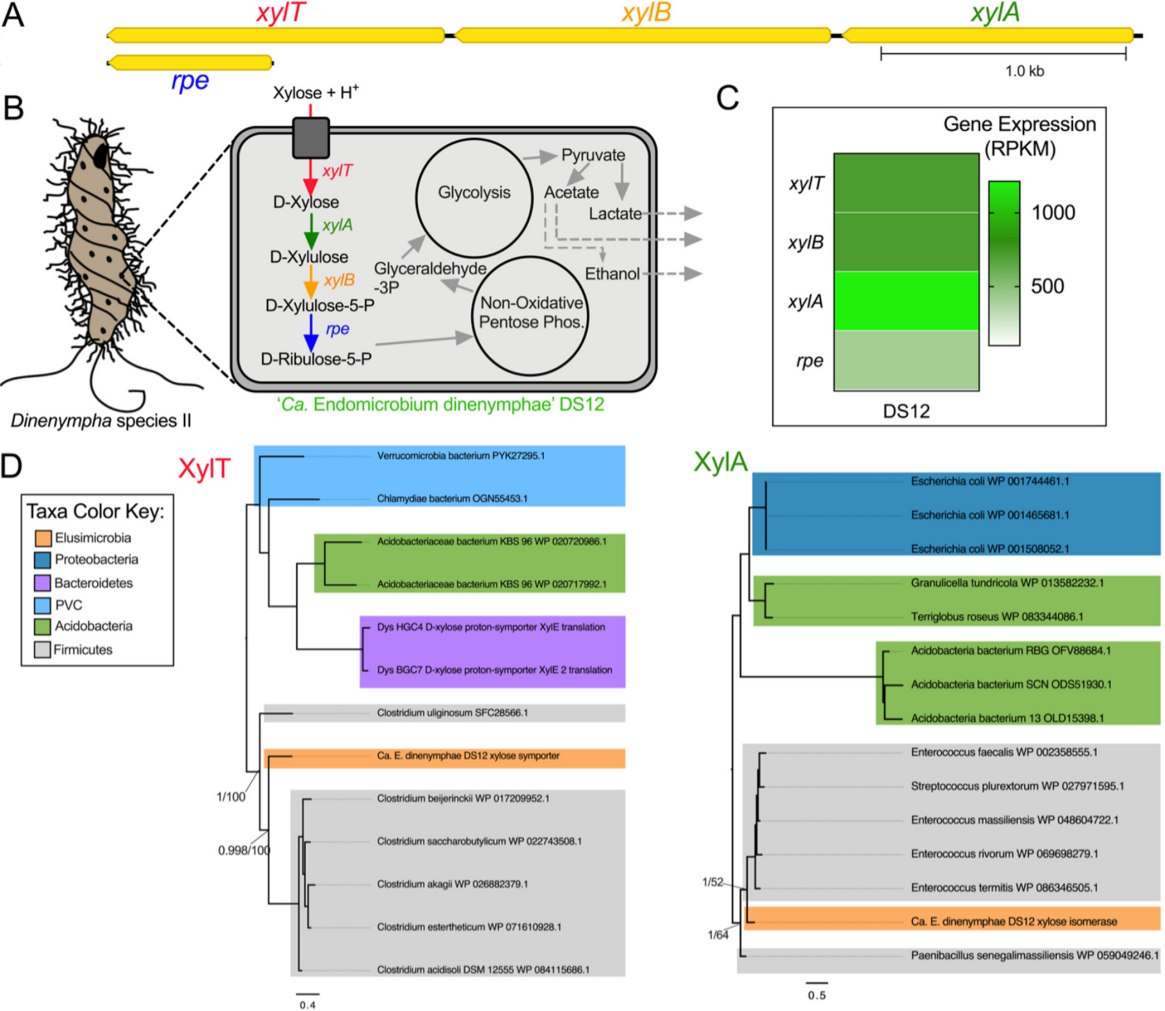
Carbon metabolism and HGT in “*Ca*. Endomicrobium dinenymphae.” (A) Gene neighborhood of the genes involved in the metabolism of xylose in the “*Ca*. Endomicrobium dinenymphae” DS12 genome. (B) Diagram of a protist host and an *Endomicrobium* cell showing the inferred metabolic conversions of carbon sources based on gene content data. (C) Gene expression data of genes of interest (rows) pertaining to carbon metabolism in “*Ca*. Endomicrobium dinenymphae” DS12 (column). (D) Maximum likelihood phylogenetic trees of amino acid sequences from the transporter (XylT, using substitution model LG+F+G4) and isomerase (XylA, using substitution model LG+G4) in the xylose metabolism pathway. Support values represent the Bayesian posterior probability and bootstrap support values, respectively.

Based on the gene content in the five genomes analyzed here, the carbon sources capable of being catabolized by endosymbiotic *Endomicrobium* species may often differ from each other and from their free-living relatives. For example, “*Ca.* Endomicrobium agilae” TA21 and TA26 encoded all the genes necessary to import and use both glucuronate and glucose-6-phosphate (Fig. 3A and 3B). The closely related “*Ca*. Endomicrobium trichonymphae” Rs-D17 (3) also contained these genes. Interestingly, genome analyses suggest that these two carbon sources cannot be used by the other *Endomicrobium* species studied here which lack genes encoding the glucuronate transporter ExuT, the glucuronate isomerase UxaA, and the glucose-6-phosphate transporter UhcP. The other *Endomicrobium* genomes encoded either arabinose (“*Ca*. Endomicrobium pyrsonymphae” PV1 and PV7) or xylose (“*Ca*. Endomicrobium dinenymphae” DS12) import and catabolism proteins that were not encoded in the TA21, TA26, *E. proavitum*, or “*Ca*. Endomicrobium trichonymphae” Rs-D17 genomes. (Fig. 4 and 5, respectively). Transcriptome data indicated that each of the genes involved in these carbon usage pathways were expressed in the respective *Endomicrobium* while they resided in their protist hosts (Fig. 3C, 4C, and 5C). Metabolites from these carbon sources are typically fed into both the nonoxidative pentose phosphate pathway and glycolysis, of which both are complete in the five genomes described here (Fig. 3B, 4B, and 5B; Fig. S2C).

Other likely differences in the carbon metabolism of these *Endomicrobium* species included the production of fermentation end products (Fig. S2C). An analysis of the five *Endomicrobium* genomes suggested that following glycolysis, pyruvate can be fermented to acetate; however, only the genomes of “*Ca.* Endomicrobium agilae” and “*Ca*. Endomicrobium dinenymphae” encoded AdhE, which can convert acetate to ethanol. In addition, genes encoding lactate dehydrogenase (Ldh) were in the genomes of both “*Ca*. Endomicrobium dinenymphae” and “*Ca*. Endomicrobium pyrsonymphae” but not in “*Ca.* Endomicrobium agilae” or “*Ca*. Endomicrobium trichonymphae” Rs-D17 (Fig. 3B, 4B, and 5B). Differences in these fermentation pathways between free-living *E. proavitum* and “*Ca*. Endomicrobium trichonymphae” Rs-D17 were described earlier by Zheng et al. (18).

Previous studies identified genes acquired by horizontal gene transfer (HGT) in other *Endomicrobium* species (18); therefore, we tested whether HGT could, at least in part, explain the differences seen in carbon metabolism across the genome sequences presented in this study. Phylogenetic trees were made for each of the transport and isomerase proteins in the glucuronate, arabinose, and xylose degradation pathways (Fig. 3D, 4D, and 5D), and the phylogenies were compared with the *Endomicrobium* 16S rRNA gene phylogeny to determine if they were congruent (see Fig. S4 in the supplemental material). In each case, these phylogenies were not congruent, suggesting that these genes were acquired by HGT (Fig. 3D, 4D, and 5D). Likely donor taxa include *Bacteroidetes*, *Actinobacteria*, and *Firmicutes* (Fig. 3D, 4D, and 5D), which are all part of the hindgut community of *R. flavipes* (8). An additional analysis of these genes by HGTector (25) indicated that they were likely acquired by HGT from the same donor taxa that were suggested by the phylogenies (Tab6 in Data Set S1). Similar data regarding HGT in “*Ca*. Endomicrobium trichonymphae” Rs-D17 have been reported and suggest that *Endomicrobium* symbionts are not cut off from gene flow and HGT (18). This finding contrasts with information known about the endosymbionts of sap-feeding insects, which are traditionally thought to experience little to no gene flow; however, recent analyses suggested that HGT may occur more frequently than previously thought in these symbionts (26).

10.1128/msphere.00021-22.6 FIG S3 Mapping coverage of the histidine biosynthesis pathway in “*Ca*. Endomicrobium pyrsonymphae” PV1. Metagenomic reads from sample PV1 were mapped to the draft genome of “*Ca*. Endomicrobium pyrsonymphae PV7.” The resulting coverage of these genes indicate that “*Ca*. Endomicrobium pyrsonymphae PV1” carried the histidine biosynthesis pathway and that the reason those genes are missing from the draft genome is likely due to an artifact of assembly or binning. Download FIG S3, JPG file, 1.5 MB. Copyright © 2022 Stephens et al. 2022 Stephens et al. https://creativecommons.org/licenses/by/4.0/ This content is distributed under the terms of the Creative Commons Attribution 4.0 International license.

10.1128/msphere.00021-22.7 FIG S4 16S rRNA phylogeny of *Elusimicrobia* with respect to other phyla. Maximum likelihood phylogenetic tree of 16S rRNA genes (using substitution model TPM3u + G4) which was used a marker gene to establish an organismal phylogeny of the *Elusimicrobia* phylum. This phylogeny was used to determine incongruence between the 16S rRNA gene and other genes of interest that may have been acquired via HGT by the *Endomicrobium* species. Download FIG S4, JPG file, 0.9 MB. Copyright © 2022 Stephens et al. 2022 Stephens et al. https://creativecommons.org/licenses/by/4.0/ This content is distributed under the terms of the Creative Commons Attribution 4.0 International license.

### Natural transformation and competence as a possible mechanism for acquiring genes.

Analyses of sequenced genomes of endosymbiotic *Endomicrobium* lineages imply that the acquisition of genes by HGT is relatively common. Interestingly, compared with other endosymbionts, the *Endomicrobium* genomes were enriched in genes related to the uptake of exogenous DNA and recombination (natural transformation/competence) (see Fig. S5 in the supplemental material). Of special interest are the *Endomicrobium* genes *comEC*, *comEB*, *comF*, *comM*, *ssb*, *drpA*, and *recA* which are all involved in natural transformation in bacteria, such as Vibrio cholerae (27).

10.1128/msphere.00021-22.8 FIG S5 Gene content regarding genes involved in natural transformation, pilus assembly, and DNA recombination/repair. (A to C) Presence and absence matrices of genes involved in natural transformation (A), pilus assembly (B), and DNA repair and recombination (C) found the endosymbiotic *Endomicrobium* genomes. The presence of these genes was investigated in their free-living relatives (Rsa215 and *E. minutum* Pei191) and other endosymbiotic bacteria. Accession numbers for references genomes used in this analysis are provided in Tab4 of Data Set S1. Note that the genes marked with a “*” or a “!” were not in the final assemblies, but their reads were detected by either MEGAN (!) or by read mapping onto the same gene from another closely related organisms (*) or both (!*) (see Fig. S4 as an example). Download FIG S5, JPG file, 0.4 MB. Copyright © 2022 Stephens et al. 2022 Stephens et al. https://creativecommons.org/licenses/by/4.0/ This content is distributed under the terms of the Creative Commons Attribution 4.0 International license.

**FIG 6:**
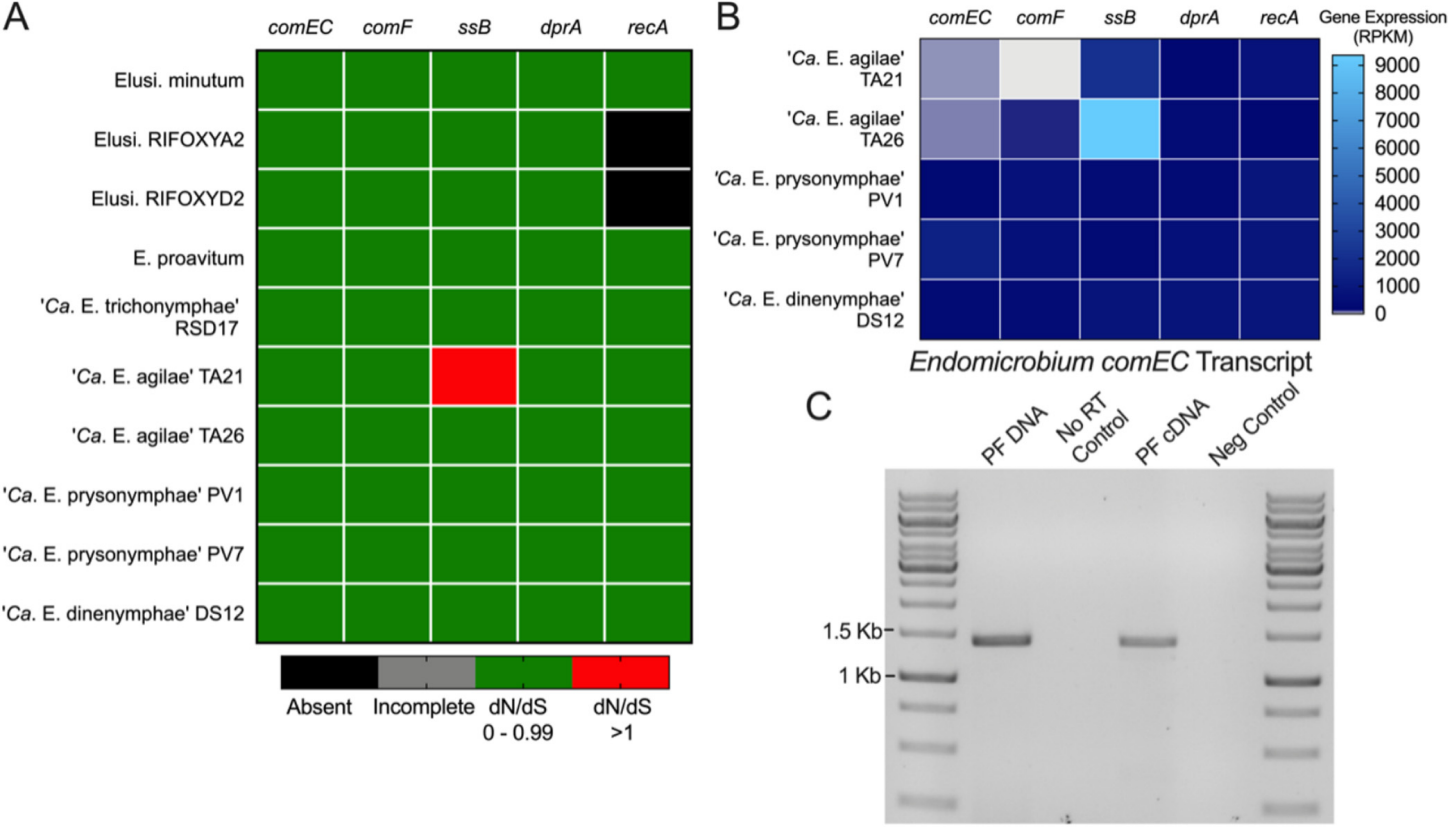
An analysis of the genes involved in a putative competence pathway in *Endomicrobium* spp. (A) Heatmap showing the results of *dN*/*dS* analyses of genes involved in competence and recombination (columns) from *Endomicrobium* spp. and *Elusimicrobium* relatives (rows). (B) Gene expression data of those genes (columns) in the *Endomicrobium* spp. (rows) presented in this study. (C) RT-PCR gel image of *Endomicrobium comEC* transcript. Samples consisted of protist fraction (PF) DNA (positive control), no-RT control, PF cDNA, and molecular-grade water (negative control). Accession numbers for reference genomes used can be found in Tab2 of Data Set S1.

The ratio of nonsynonymous to synonymous evolutionary changes (*dN*/*dS* ratio) of these genes supported the hypothesis that selection was acting to maintain the amino acid sequences of their corresponding gene products (*dN*/*dS* ratio, <1.0), with the exception of *ssb* from TA21 (Fig. 6A). In addition, a transcriptome analysis indicated that these genes were expressed (Fig. 6B). Expression of *comEC*, which encodes a transporter that imports single-stranded DNA across the inner membrane and into the cytoplasm of Gram-negative bacteria (27, 28), was verified in “*Ca*. Endomicrobium agilae” by reverse transcriptase PCR (RT-PCR) and sequencing by using *comEC*-specific primers on a protist cell fraction sample prepared from 20 worker termite hindguts (Fig. 6C). Together, these data support the hypothesis that genes involved in this competence pathway are both conserved and expressed in these *Endomicrobium* symbionts of hindgut protists of *R. flavipes*.

The competence genes discussed above are involved in the translocation of single-stranded DNA across the inner membrane of Gram-negative bacteria and subsequent recombination. Also present in the genomes of all five *Endomicrobium* species analyzed in this study are genes which encode proteins that are similar to type IV pilins. Some pilins from classes type IV and type II can bind and import double-stranded DNA across the outer membrane and have been shown to work in conjunction with ComEC-type proteins (27, 29). The TA21 and TA26 genomes contained a large chromosomal region devoted to type IV Tad-like pilus synthesis as does *E. proavitum* and Elusimicrobium minutum. The bacterium “*Ca*. Endomicrobium trichonymphae” Rs-D17 has a similar region, but it appears that many of the genes have become pseudogenes. The *P. vertens* and *Dinenympha* species II *Endomicrobium* symbionts had genes encoding pilins similar to the type II PulG pilins. In addition, all five genomes possessed a prepilin peptidase (PilD) (Fig. S5). A graphical summary of these findings along with a model of how competence may work in these organisms is provided as Fig. S6 in the supplemental material. A list of genes and their putative function can be found in Tab10 and Tab11 of Data Set S1.

10.1128/msphere.00021-22.9 FIG S6 Graphical summary of a putative competence pathway and proteins involved in pilus assembly in *Endomicrobium* species. Proteins shared by all *Endomicrobium* species are in black font, while those that are retained only in “*Ca*. Endomicrobium agilae” are colored in orange. All *Endomicrobium* possessed the prepilin peptidase (PilD) and one or more genes that encode pilins. “*Ca*. Endomicrobium agilae” possessed a near-complete *tad* locus/type 2 secretion system (T2SS) as well the type IV pilin (T4P) secretin (PilQ). Download FIG S6, JPG file, 0.7 MB. Copyright © 2022 Stephens et al. 2022 Stephens et al. https://creativecommons.org/licenses/by/4.0/ This content is distributed under the terms of the Creative Commons Attribution 4.0 International license.

### Transcriptome analysis of *Endomicrobium* populations inside single protist cells.

Reads from each of the five single-cell metatranscriptomes were then mapped to all contigs in their matching metagenome and to their respective *Endomicrobium* draft genome. For the vast majority of *Endomicrobium* genes, the reads per kilobase per million (RPKM) values were the similar for both mappings, indicating most *Endomicrobium* reads did not match contigs that were not part of *Endomicrobium* draft genomes (Tab7 of Data Set S1).

**FIG 7:**
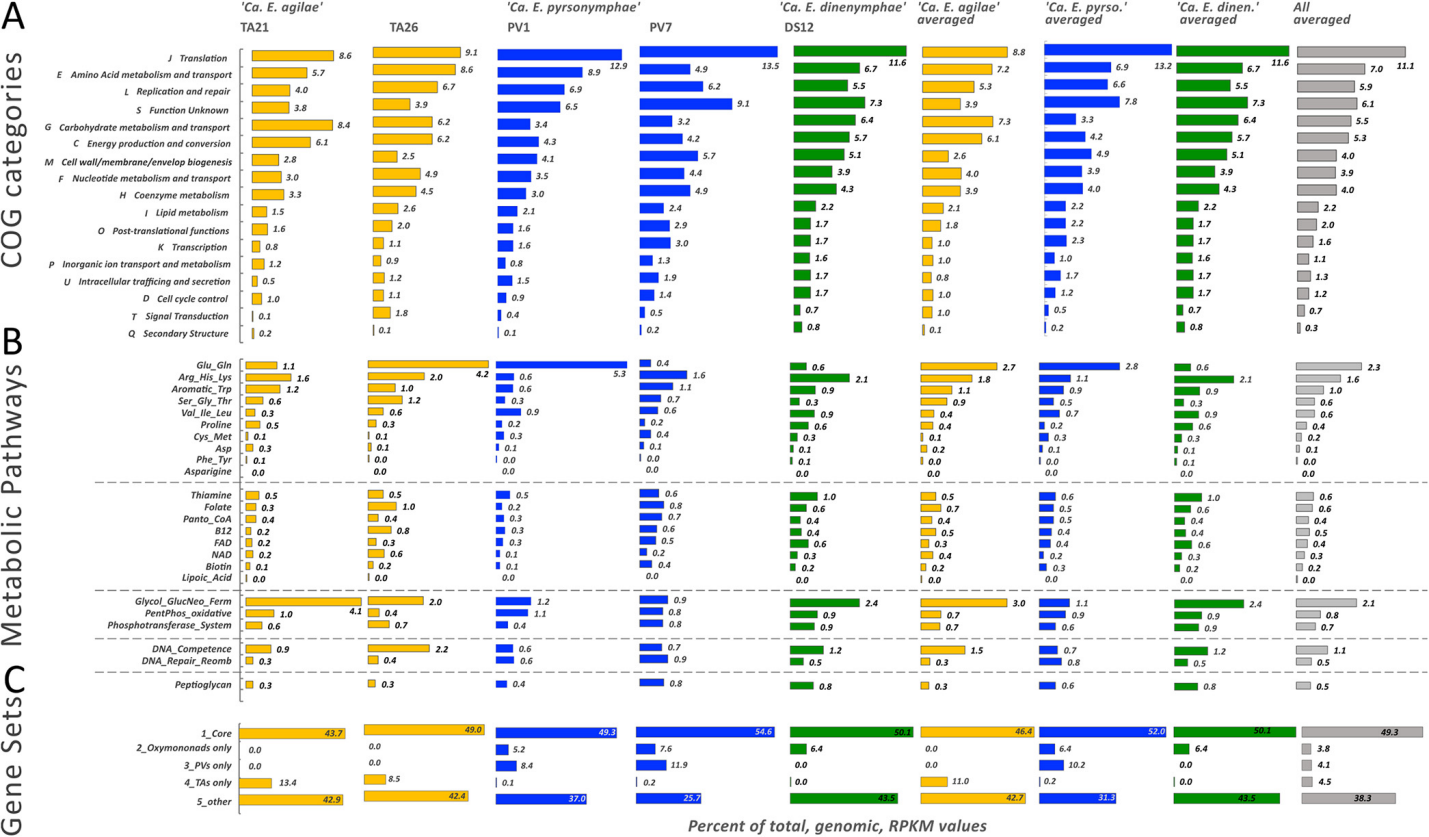
Transcriptome analysis of *Endomicrobium* populations from individual protist cells. (Top) Expression analysis of genes grouped into Clusters of Orthologous Groups (COG) functional categories. (Middle) Expression analysis of genes in certain metabolic pathways pertaining to amino acid and cofactor biosynthesis, DNA processing, and peptidoglycan biosynthesis. (Bottom) Expression of genes grouped by their distributions among the different *Endomicrobium* species. RPKM values for individual cells and averages of genera and all cells are in the various columns.

Transcriptome analyses of the *Endomicrobium* populations inside single protist cells (from which the five genomes were derived) revealed similar gene expression profiles with a few notable exceptions (Fig. 7). While our sample size for this work was necessarily small and while we were unable to do time-resolved sampling of the hindgut community from single termites, some general trends did appear in the transcriptomic data. Among Clusters of Orthologous Groups (COG) categories, which are quite broad, the expression by each endosymbiont population was relatively similar with one exception being that there was a higher expression of genes related to carbohydrate transport and metabolism in *Trichonympha* hosts and *Dinenympha* (TA21, TA26 and DS12) than that of the *Pyrsonympha* hosts (PV1 and PV7) (Fig. 7A).

Our analyses which focused on narrower categories, such as genes in related biosynthetic pathways, carbohydrate transport and break down, peptidoglycan synthesis, and DNA uptake and repair, revealed further differences not only between the endosymbionts of different protist species but also between the populations of endosymbionts of individual protist cells of the same type (Fig. 7B). These expression differences between bacterial populations in the same protist cell type are demonstrated by the differences related to the expression of genes in the glutamine and glutamate biosynthesis pathway (Fig. 7B). Overall, this pathway is more highly expressed by the endosymbionts of *Pyrsonympha* hosts than that of endosymbionts in other protist species, but there was variation in the expression of this pathway between individual protist host cells. For example, in host cell PV1, this pathway represented 5.3% of the total transcriptome reads, mostly from the gene *glnN* encoding a glutamine synthase, whereas in host cell PV7, it comprised only 0.4% (Fig. 7B). A similar variation in this pathway was seen in the TA21 and TA26 transcriptomes. These data suggest that even populations of the bacterium residing in different host cells may not be expressing the same functions at any given point in time.

Core genes, which were shared between all five of the *Endomicrobium* species, represented an average of 30% to 36% of the transcripts of each endosymbiont population (Fig. 7C). The expressions of genes that were specific to each *Endomicrobium* species ranged from 11% in “Ca. Endomicrobium pyrsonymphae” to 22% in “Ca. Endomicrobium agilae,” indicating that there is differential gene content and gene expression of endosymbionts of different protist host species (Fig. 7C). Transcriptomic data are in Tab7 and Tab8 in Data Set S1.

## DISCUSSION

Single-cell protist metagenomics has enabled the assembly of genome sequences from several protist-associated bacterial symbionts from termite hindguts (1, 3, 4, 30, 31). In this study, we present near-complete draft genome and transcriptome sequences of five endosymbiotic *Endomicrobium* samples, from three different protist species. Those endosymbionts displayed differences with regard to their gene content and its expression. For example, these organisms possessed different carbon usage pathways. One hypothesis that may explain such differences in carbon utilization is that different by-products from protists hydrolyzing and fermenting wood may be provided as carbon sources to the *Endomicrobium* endosymbionts. For example, glucuronate may be present in the cytoplasm of *Trichonympha* spp. because they possessed the enzymes needed to cleave those monomers from polysaccharides found in wood, whereas the other protists, *P. vertens* and *D*. species II, may not be able to generate such monomers, or they may use them for other purposes (see below). If true, this hypothesis suggests that there may be specialization among the protists with regard to polysaccharide hydrolysis in the hindgut of *R. flavipes* A recent study demonstrated a division of labor among symbiotic protist species in a different termite, namely, Coptotermes formosanus. There, protist species produced different hydrolytic enzymes to degrade polysaccharides found in wood (32). These differences in protist functions may also explain why there was a higher expression of genes related to carbohydrate transport and metabolism in the endosymbionts of *Trichonympha* hosts than that of *Pyrsonmympha* hosts (Fig. 7).

However, an alternative hypothesis is that metabolites can be partitioned within the host and some are specifically provided to certain symbionts. This hypothesis, if true, may allow the host to control endosymbiont population densities through selective carbon source provision. Such host control of carbon provisioning is thought to operate in nitrogen-fixing root nodule symbioses ensuring that bacterial symbionts continue to provide fixed nitrogen in return for plant-provided carbon. In support of this second hypothesis, the membrane-embedded symbiont “*Ca*. Desulfovibrio trichonymphae,” which cocolonizes the same *Trichonympha* host as “*Ca*. Endomicrobium trichonymphae” Rs-D17, uses malate and citrate as carbon sources, whereas its coinhabitant Rs-D17 likely uses glucuronate and glucose-6-phosphate (31).

Evidence suggests that *Endomicrobium* species have acquired genes by HGT and some of the donor taxa may include termite-associated bacteria. Endosymbiotic lineages of *Endomicrobium*, and their free-living relatives, possess many genes involved in DNA uptake, repair, and recombination (Fig. S5). Our analyses showed that the genes *comEC*, *comEB*, *comF*, *comM*, *ssb*, *drpA*, and *recA* are usually conserved within the *Elusimicrobia* phylum and were expressed in the endosymbiotic *Endomicrobium* species characterized in this study (Fig. 6). Collectively, these genes have been shown to be involved in the translocation of single-stranded DNA across the inner membrane of other Gram-negative bacteria and in homologous recombination (27). The gene *comEC*, in particular, has an important function in this process, as it encodes an essential part of the DNA transporter (27, 28). Using transcriptome data, RT-PCR, and sequencing, we were able to show that the “*Ca*. Endomicrobium agilae” *comEC* gene is expressed in these endosymbionts (Fig. 6). Collectively, the genes involved in the competence pathway comprised between 0.4% and 1.3% of the total transcriptome reads of each endosymbiont population (Fig. 7). These data suggest that *Endomicrobium* species may have the ability to become competent which may allow them to acquire DNA from the wider termite gut community and could result in HGT.

It is not clear how these organisms transport DNA across their outer membranes. None of these *Endomicrobium* species possessed all the components of a type IV pili-based DNA-translocation system, but “*Ca*. Endomicrobium agilae” TA21 and TA26 contained a near-complete type IV *tad* system which may allow DNA uptake (Fig. S5 and 6). It is also puzzling why each of the five genomes has retained *pilD*, a prepilin peptidase, as well as genes encoding type II and type IV pilins. They may carry out some function in DNA uptake or they may be nonfunctional and are in the process of being lost. If competence is a common trait among the *Endomicrobium* members, it could explain why these organisms have many genes acquired through HGT, and this capability may allow for their rapid adaptation to new and diverse niches. Because hindgut protists phagocytize wood, wood-associated bacteria, and perhaps free-living hindgut bacteria (33), it may be a route through which endosymbiotic *Endomicrobium* members could be exposed to exogenous DNA.

However, it is worth noting that competence is not the only plausible avenue for DNA acquisition in these endosymbionts. HGT could also occur by bacteriophage transduction, conjugation, or other routes. Several lines of evidence indicate that these endosymbionts are susceptible to molecular parasites, such as bacteriophages and plasmids. Previous studies have reported that *Endomicrobium* species possessed several intact defense mechanisms to combat molecular parasites, such as CRISPR-Cas and restriction-modification systems (34, 35). The *Endomicrobium* species sequenced in this study also contained those defense systems. The complete genome sequence of a bacteriophage of an endosymbiont (“*Candidatus* Azobacteroides pseudotrichonymphae”) of a termite hindgut protist has been published previously, indicating that phage infection is not limited to *Endomicrobium* endosymbionts and may be common in termite hindguts (36).

Our analysis of *Endomicrobium* genomes and transcriptomes obtained from single protist cell metagenomes highlight important differences between protist hosts which have led to hypotheses that warrant further investigation. In each case, the major hurdle of testing these hypotheses is the current inability to culture the protist hosts, which restricts their experimental tractability. However, the use of additional approaches should further our understanding of these symbioses by focusing on the host and their symbiont genes, mRNA, and metabolic and protein contents (37–42).

## MATERIALS AND METHODS

### Termite collection and species identification.

*R. flavipes* termites were collected using cardboard traps placed under logs for 2 to 4 weeks at the UConn Campus at Storrs, Connecticut (longitude, −72.262216; latitude, 41.806543), and their identity was verified as described previously (7) by amplifying and sequencing the mitochondrial cytochrome oxidase II gene. Termites were maintained in the lab with moistened sand and spruce wood that were initially sterilized.

### Single protist cell isolation.

Termites from the worker caste were brought into an anaerobic chamber, and their hindguts were dissected with sterile forceps. Hindguts were ruptured in ice-cold Trager’s solution U (TU) (43) and washed three times by centrifuging in 500 μL of TU at 3,000 rpm in an Eppendorf microcentrifuge for 90 seconds. This washed cell suspension was then diluted 10-fold in TU buffer on ice. A 1-μL aliquot of the washed and diluted cell suspension was added to a 9-μL droplet on a glass slide treated with RNase Away reagent (Life Technologies) and UV light. Individual protist cells were isolated using a micromanipulator (Eppendorf CellTram Vario) equipped with a hand-drawn glass capillary. Individual cells were washed three times in 10-μL droplets of TU via micromanipulation, transferring approximately 0.1 μL each time, and finally placed in 10 μL molecular grade phosphate-buffered saline (PBS), flash frozen on dry ice, and immediately stored at −80°C.

### Whole-genome and transcriptome amplification and sequencing.

The metagenome (DNA) and metatranscriptome (cDNA) from individual protist cells and their associated bacteria were amplified simultaneously at 12 to 24 hours after isolation. Cell lysis and amplification were performed using the Repli-g whole-genome amplification/whole-transcriptome amplification (WGA/WTA) kit (Qiagen). Cells were lysed using a Qiagen lysis buffer followed immediately by incubation on ice. Two samples from each lysed cell were taken and used for whole-genome amplification and whole-transcriptome amplification. These amplifications were carried out using the manufacturer’s standard protocol with the exception that random hexamer primers were used to amplify DNA and cDNA. DNA and cDNA were sheared using a Covaris M220 ultrasonicator according to the manufacturer’s protocol. WGA samples were sheared to a 550-bp insert size using 200 ng of DNA. WTA samples were sheared to a 350-bp insert size using 100 ng of cDNA. Sequencing libraries were prepared using the TruSeq Nano DNA library prep kit from Illumina according to the manufacturer’s protocol. Each sample was prepared with a forward and reverse barcode such that samples could be multiplexed on the same sequencing run. The samples were sequenced using an Illumina NextSeq 1 × 150-bp midoutput run and two NextSeq 1 × 150-bp high-output runs. Metadata regarding WGA/WTA yields can be found in Tab1 in Data Set S1.

### Genomic read processing and assembly.

Reads were preprocessed before assembly using BBMap (44). Reads were filtered for contaminating sequences by mapping reads to reference genomes of potential contamination sources, such as human DNA, human-associated microbiota, and organisms used commonly in our research laboratories. A list of reference genomes used for contamination filtering is provided in Tab2 in Data Set S1. Using BBMap scripts, adaptor sequences were trimmed from reads and the last base pair of 151-bp reads was removed. Reads were then trimmed at both ends using a quality score cutoff of Q15. Homopolymers were removed by setting an entropy cutoff of 0.2 and a max G+C cutoff of 90% and by removing reads which possessed stretches of Gs equal to or greater than 23 bases long. In addition, reads which were below a minimum average quality of Q15 and/or less than 50 bases long were removed. Genomic reads were then normalized to a minimum coverage of 2× and a maximum coverage of 50× and then deduplicated using BBnorm. Genomic reads were assembled using the A5 assembly pipeline (45) on the KBase Web server (46). Data regarding metagenome and metatranscriptome read numbers can be found in Tab3 in Data Set S1.

### Taxonomic composition of the assemblies.

In order to identify the major taxonomic groups in the metagenome assemblies, reads in the assemblies were treated as amplicon sequence variants (ASVs) and mapped against a set of 16S rRNA V4 ASVs from previous work (7) in addition to V4 ASVs from DictDb v3.0 (42), SILVA v138 (prok), and SILVA v132 (euk) (41) rRNA databases. The DictDb and SILVA V4 ASVs were selected because they differed from the in-house ASVs and at least 0.5% of reads in the five metagenomes mapped to them at greater than 98% identity. In total, there were 8 eukaryotic 18S V4 ASVs and 139 16S ASVs in this reference data set (Tab5 in Data Set S1). Reads from the metagenomes were mapped in Geneious Prime 2021 and had to match at 100% identity to a reference ASV over its entire 149- to 150-base length in order to be counted as mapped. A less stringent 98% identity cutoff was also used in order to determine if reads failed to map because our reference set was incomplete.

### Genomic binning, draft genome assessment, and annotation.

Metagenomic assemblies from single protist host cells and their bacterial symbionts were binned using either 4-mer, 5-mer, or 6-mer frequencies with VizBin (47) and scaffolds at least 1 Kb long. Clustered scaffolds in genomic bins of interest (low G+C content) were selected in VizBin (see Fig. S1 in the supplemental material). Scaffolds from these bins were used in a blastn (48) search against previously sequenced *Elusimicrobia* genomes (Tab4 in Data set S1). Scaffolds which had a positive hit to other *Elusimicrobia* (at least 70% identity over a 1-kb alignment) were retained in the draft genomes, and scaffolds which did not have a significant hit to other *Elusimicrobia* genomes were used in a second blastn search against the nonredundant (NR) database. From these searches, scaffolds which had positive hits to other *Elusimicrobia* in the NR database were retained. Draft genomes were iteratively polished with the program Pilon (49). These draft genomes were then assessed for contamination and completeness using CheckM which uses lineage-specific marker genes to perform analyses (50). The resulting near-complete draft genomes were annotated initially on the RAST server using a customized RASTtk workflow with options selected to call insertion sequences and prophages (51, 52). Metabolic pathways pertaining to carbon metabolism, amino acid biosynthesis, vitamin biosynthesis, and peptidoglycan biosynthesis were reconstructed from the annotated genomes using pathways in the Kyoto Encyclopedia of Genes and Genomes (KEGG) (53).

10.1128/msphere.00021-22.4 FIG S1 VizBin diagrams indicating contigs used in the five *Endomicrobium* draft genomes. Metagenomes were assembled as described in Materials and Methods and then binned with VizBin (47) based on 5-mer frequencies and transcriptome coverage. Only contigs larger than 2,000 bp are shown in this figure. Contigs denoted by orange stars indicate the contigs that make up each draft genome. Download FIG S1, JPG file, 0.8 MB. Copyright © 2022 Stephens et al. 2022 Stephens et al. https://creativecommons.org/licenses/by/4.0/ This content is distributed under the terms of the Creative Commons Attribution 4.0 International license.

### Analysis of ribosomal gene phylogeny and average nucleotide identities.

Ribosomal 16S genes from each of the *Endomicrobium* draft genomes were trimmed and aligned to references using MUSCLE (54), evolutionary models were tested, and a maximum likelihood (ML) phylogenetic tree was made using IQ-TREE (55). JSpeciesWS (22) was used for determining the genomic average nucleotide identities based on BLAST+ searches (ANIb) between the *Endomicrobium* draft genomes and the genome of “*Ca*. Endomicrobium trichonymphae” Rs-D17, which is a close relative (3).

Assembled 18S rRNA genes were retrieved from metagenome assemblies by performing a BLAST+ search using previously published 18S rRNA reference sequences for each protist species as queries (7). When possible, protist 18S rRNA genes were amplified using leftover DNA from WGA samples using universal primers 18SFU (5′-ATGCTTGTCTCAAAGGRYTAAGCCATGC-3′) and 18SRU (5′-CWGGTTCACCWACGGAAACCTTGTTACG-3′) (56) as described previously (7) and were sequenced by Sanger sequencing. This confirmation PCR was done on samples TA21, TA26, and DS12. Assembled 18S rRNA genes were aligned to references using MUSCLE (54), and a maximum likelihood (ML) phylogenetic tree was generated using IQ-TREE (55) with model testing. The aligned 18S rRNA sequences used for the construction of the ML tree is available in Text S1 in the supplemental material.

10.1128/msphere.00021-22.1 TEXT S1 Fasta alignment of 18S sequences. Download Text S1, TXT file, 0.1 MB. Copyright © 2022 Stephens et al. 2022 Stephens et al. https://creativecommons.org/licenses/by/4.0/ This content is distributed under the terms of the Creative Commons Attribution 4.0 International license.

### Detection of horizontally acquired genes.

Genes that may have been acquired by horizontal gene transfer were tentatively identified using HGTector (25). Briefly, the program took proteins greater than 30 amino acids long from the TA21, TA26, PV1, PV7, and DS12 genomes and compared them using Diamond BLAST (57) to a database derived from single representative species in RefSeq release 208 plus archaeal, fungal, and protist species from the NCBI Reference and Representative databases. This database contained 139,627,894 proteins from 32,128 species. HGTector used 3 *Endomicrobium* species (taxid 1408194) as the “self” data set and 11 *Elusimicrobia* species (74152) as the “close” data set. All other species were considered “distal.” In order to be considered a possible horizontally transferred gene, the test protein had to have matches in the self data set and in the distal data set but none in the close data set, as well as pass other statistical tests as outlined in Zhu et al. (25). Each query protein from the five genomes had to match with an E value of less than 1.0E-5, have a coverage of >75%, and have >50% amino acid identity to its matched target sequences in order to be considered for further analysis. These parameters and others are in the HGTector “HGTconfig.yml” and HGTector shell script file in Text S2 in the supplemental material. Putative HGT candidates and putative donor groups are included in Tab6 in Data Set S1.

10.1128/msphere.00021-22.2 TEXT S2 HGT configuration file and shell script (combined). Download Text S2, TXT file, 0.01 MB. Copyright © 2022 Stephens et al. 2022 Stephens et al. https://creativecommons.org/licenses/by/4.0/ This content is distributed under the terms of the Creative Commons Attribution 4.0 International license.

### Analysis of genes involved in competence and recombination.

Genes known to be involved in DNA uptake, competence, and recombination were identified in each *Endomicrobium* draft genome based on their NCBI-assigned annotations and homology to reference sequences. The distribution of these genes was then compared across draft genomes and references which included free-living relatives and other endosymbionts. To assess if these genes were complete and if the encoded proteins likely retained their putative functions, homologs of each gene were obtained from genomes of bacteria belonging to the phylum *Elusimicrobia* and aligned with MUSCLE (54). Phylogenetic trees were generated using IQ-TREE (55) with model testing, and support values were generated using the “–abayes” and “–bb 1000” commands. The resulting phylogenetic trees were used along with the MUSCLE (54) alignments to perform a *dN*/*dS* analysis using the program Codeml which is a part of the PAML and PAMLX packages (58, 59). Genes likely involved with competence and DNA repair are in Tab10 in Data Set S1.

### Mapping transcriptome reads to draft genomes.

Transcriptome sequencing (RNA-seq) metatranscriptome reads were quality trimmed and filtered as described above and error corrected in Geneious R11 (60) using BBNorm with default settings. To remove rRNA reads before mapping, rRNA sequences were identified from each metagenome assembly using RNAmmer (61); reads were mapped to these as well as rRNA references from RefSeq (62), SILVA (63), and DictDb (64) databases using BBMap (44). Remaining metatranscriptome reads were then mapped to all contigs in their matching metagenome and to the respective *Endomicrobium* draft genome. These mappings were done in Geneious R11 using Bowtie 2 (65) with alignment type set to “End to End” and using the “Medium Sensitivity” preset. Expression levels were then calculated in Geneious R11; ambiguously mapped reads were excluded from the calculations. RPKM values for genes in from each mapping are given in Tab7 and Tab8 in Data Set S1.

### Verification of *comEC* expression by RT-PCR.

Primers were designed to amplify *comEC* from “*Ca*. Endomicrobium agilae” in Geneious R11 using Primer3 (66) (primers were endo_comec_F [5′-ATTTGCCTGTGTTTGAGAGT-3′] and endo_comec_R [5′-CCTGTTCCTGTGCTTTCAG-3′]). Twenty termites were used to prepare RNA and cDNA samples for RT-PCR analysis. Termite hindguts were dissected and ruptured in TU on ice in an anaerobic chamber. Hindgut contents were washed with ice-cold TU three times at 3,000 rpm in an Eppendorf microcentrifuge for 90 seconds and then lysed in 1 mL of TRIzol reagent (Thermo Fisher Scientific). RNA was isolated per the manufacturer’s protocol and treated with Turbo DNase (Thermo Fisher Scientific) following the manufacturer’s protocol for 50-μL reactions using ∼5.6 mg of total RNA in a 20-μL volume. A total of 20 μL of the DNase-treated RNA was then used as the template for cDNA synthesis using SuperScript IV reverse transcriptase (Thermo Fisher Scientific) following the manufacturer’s protocol for first-strand synthesis primed with random hexamers. The resulting cDNA was treated with Escherichia coli RNaseH for 20 minutes at 37°C.

RT-PCRs were performed using the *Endomicrobium comEC* primers with RNaseH-treated cDNA serving as the template and the no-RT control consisting of DNase-treated RNA that did not undergo cDNA synthesis. RT-RCR was performed using Phusion polymerase (Thermo Fisher Scientific) with high-fidelity (HF) buffer and dimethyl sulfoxide (DMSO). Cycling conditions were as follows: initial denaturation at 94°C for 3 minutes, followed by 35 cycles of 94°C for 45 seconds, annealing at 59°C for 30 seconds, and extension at 72°C for 45 seconds. Final extension was done 72°C for 10 minutes. Hindgut DNA (washed protist cell fractions from five hindguts in molecular-grade Tris-EDTA buffer) was used as a positive PCR control. RT-PCR products were visualized using a 1% agarose gel with ethidium bromide. Products were purified using the Monarch DNA gel purification kit (New England BioLabs) and Sanger sequenced.

### Data availability.

Raw reads and assemblies were submitted to NCBI GenBank under BioProject PRJNA644342 (whole-genome assemblies are JAIXMW01, JAIXMX01, JAIXMY01, JAIXMZ01, and JAIXNA01).

10.1128/msphere.00021-22.3 TABLE S1 Taxonomy of single protist metagenome assemblies. Percentage values indicate the percentage of mapped reads from each metagenomic assembly that mapped to the indicated reference V4 ASV. Reads mapped over their whole length (149 to 150 bases) to each reference V4 ASV at greater than 98% identity with 2% gaps allowed (gaps max =3 bases). ASVs are grouped by type. Red text indicates the most common ASV for a taxon type in each metagenomic assembly. Green, yellow and grey highlighting indicate high (100% to 2%), low (<2%) and zero percent mapping values. Sequences and full names of the ASVs are in Tab5 of Data Set S1. Download Table S1, DOCX file, 0.08 MB. Copyright © 2022 Stephens et al. 2022 Stephens et al. https://creativecommons.org/licenses/by/4.0/ This content is distributed under the terms of the Creative Commons Attribution 4.0 International license.
