# Supplementary figures and images for: Simultaneous Single-Cell Genome and Transcriptome Sequencing of Termite Hindgut Protists Reveals Metabolic and Evolutionary Traits of Their Endosymbionts
Source: mSphere. 2022 Feb 2;7(1):e00021-22. doi: 10.1128/msphere.00021-22 (PMC8809381; doi:10.1128/msphere.00021-22)

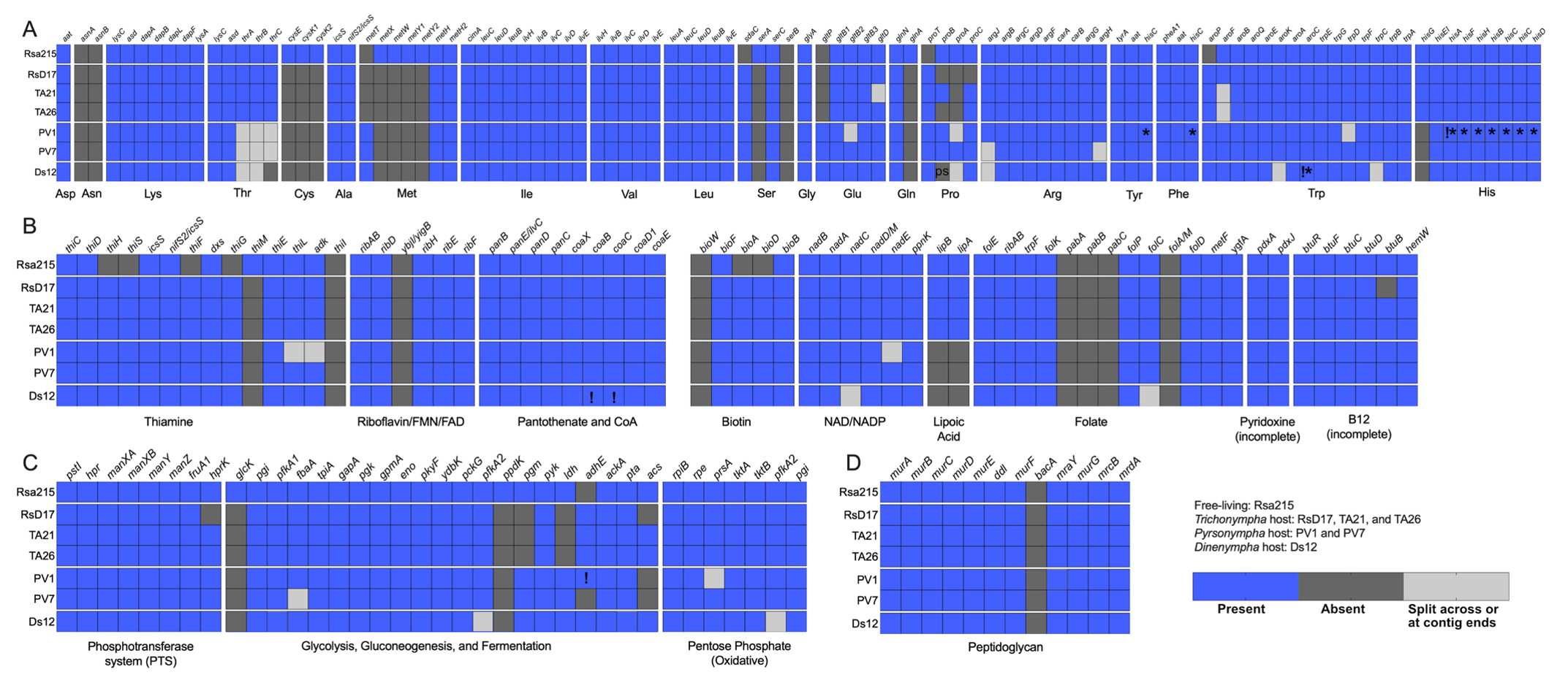

Supplement: FIG S2 [file msphere.00021-22-sf002.jpg]

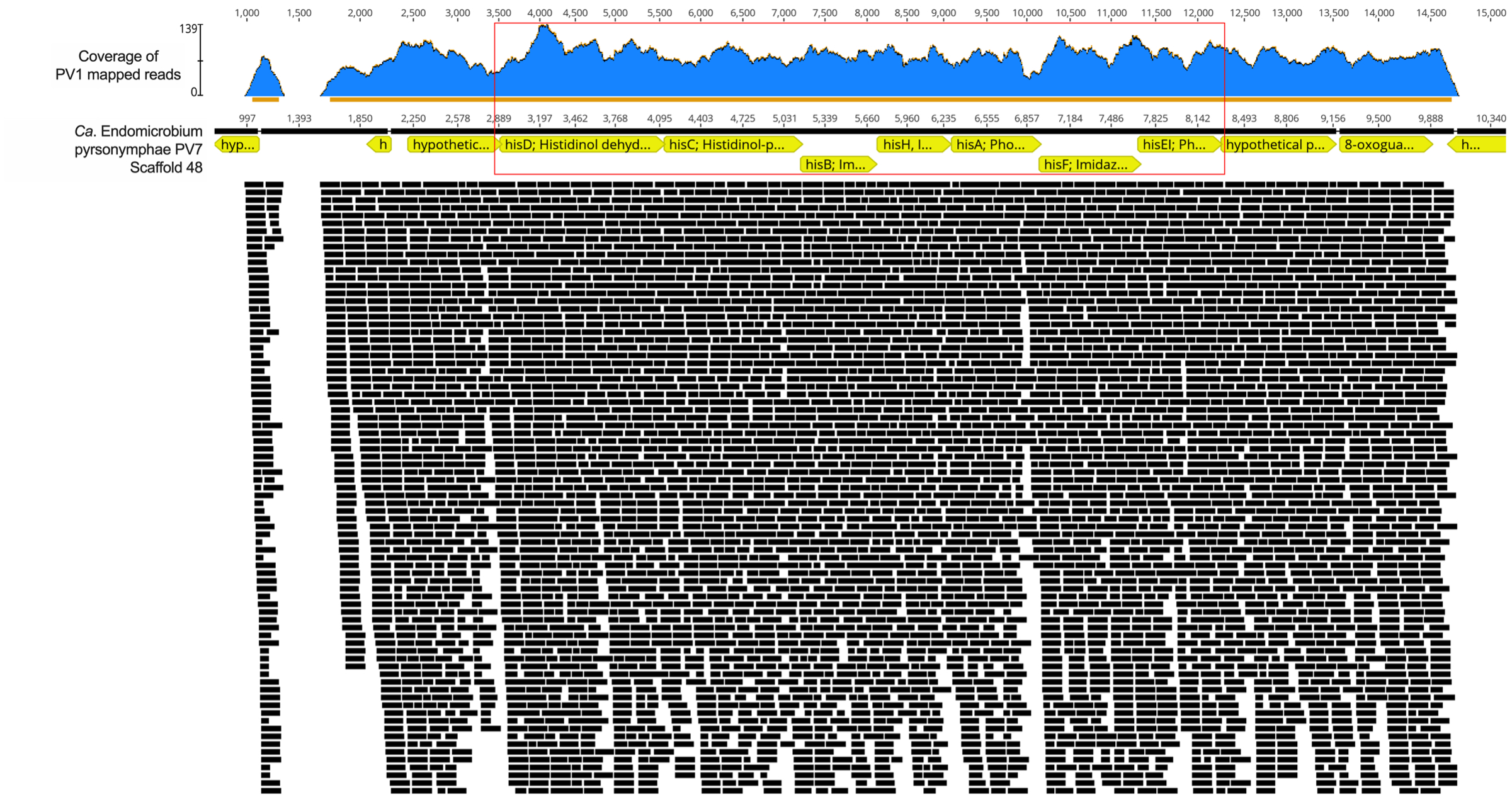

Supplement: FIG S3 [file msphere.00021-22-sf003.jpg]

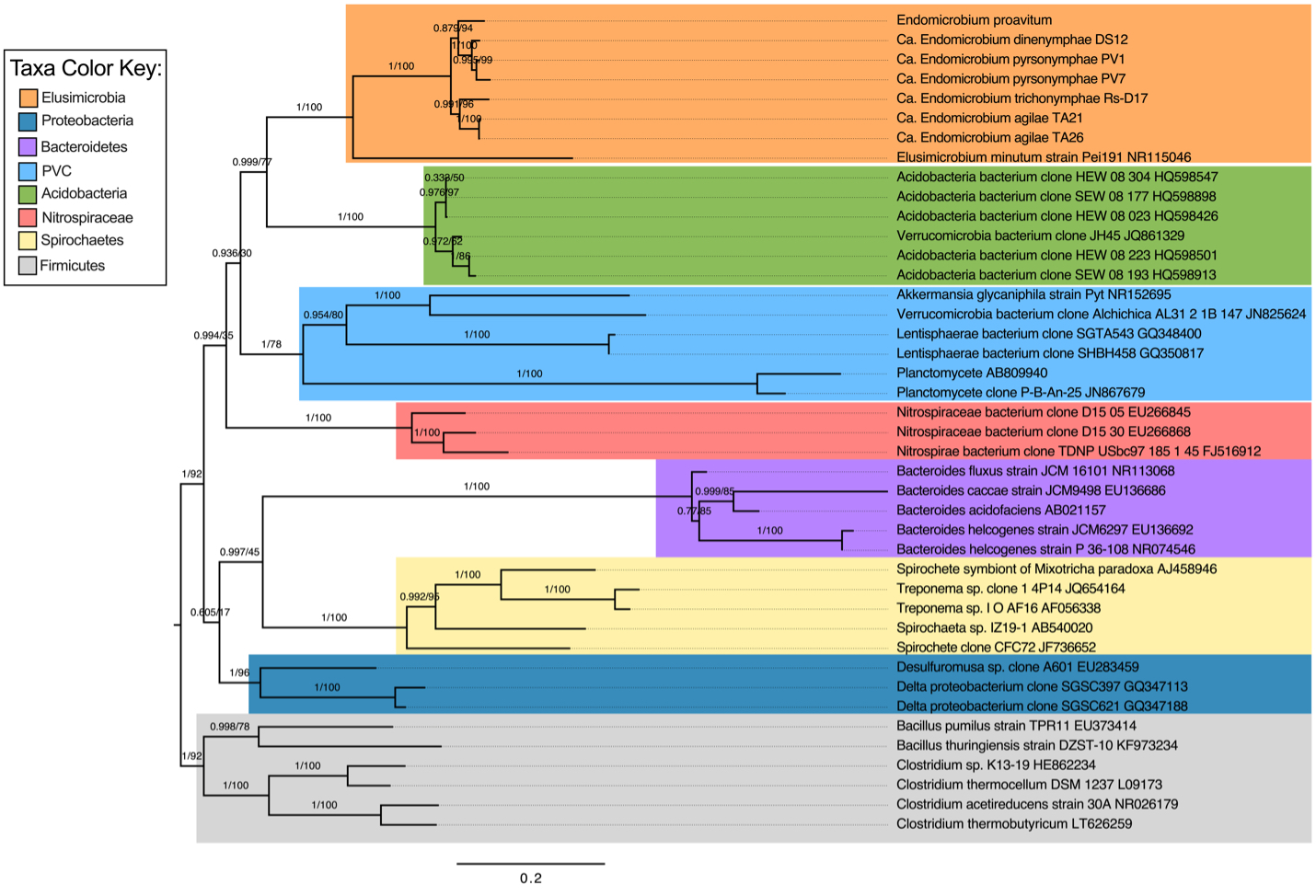

Supplement: FIG S4 [file msphere.00021-22-sf004.jpg]

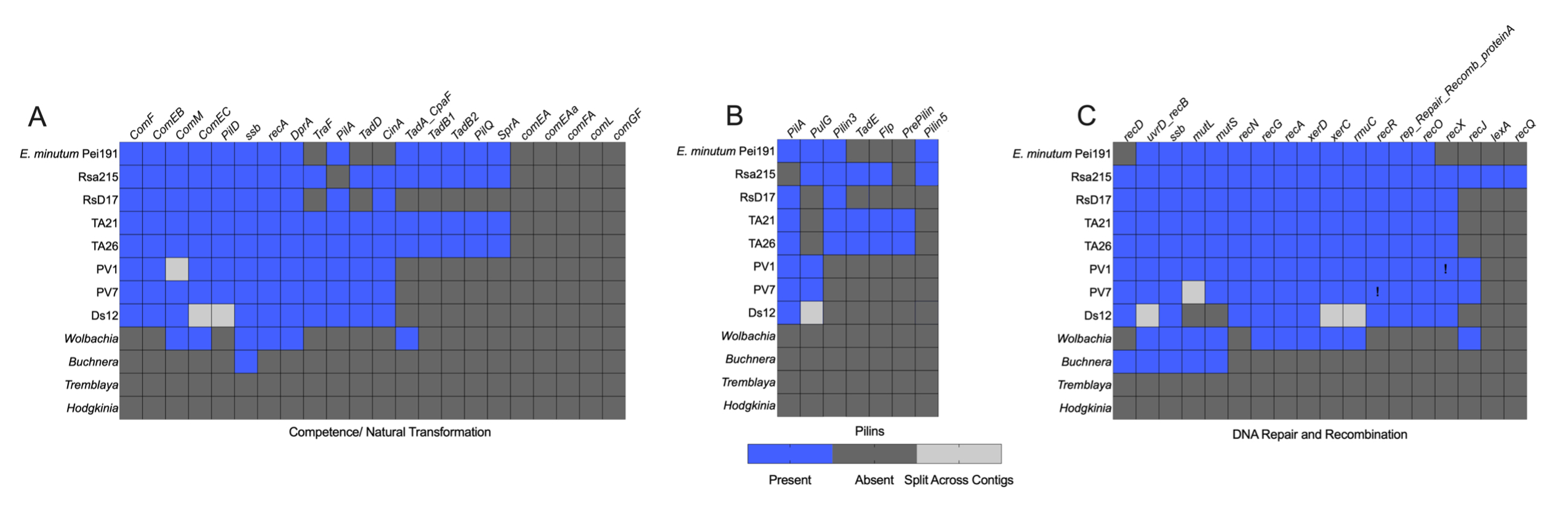

Supplement: FIG S5 [file msphere.00021-22-sf005.jpg]

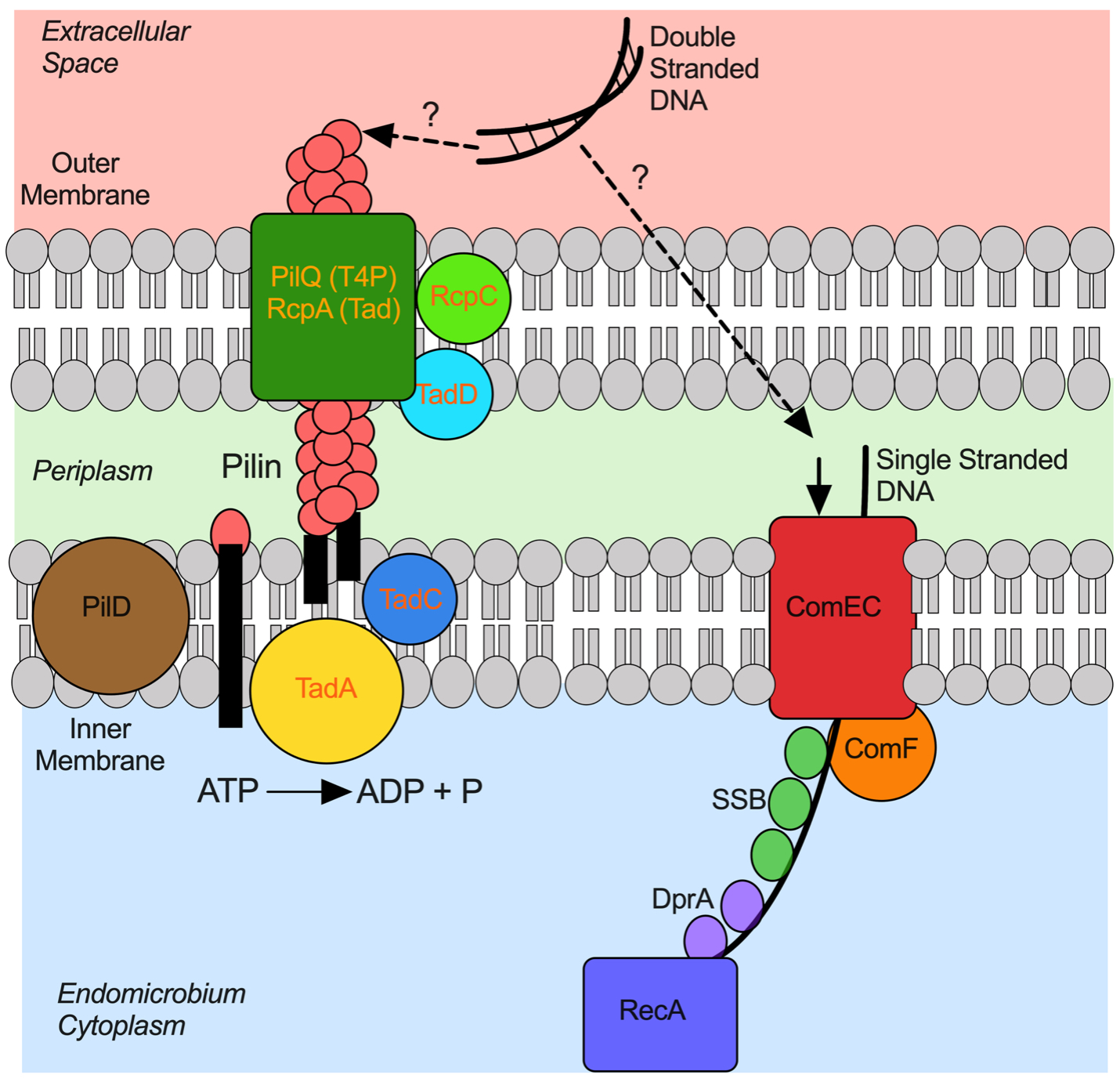

Supplement: FIG S6 [file msphere.00021-22-sf006.jpg]

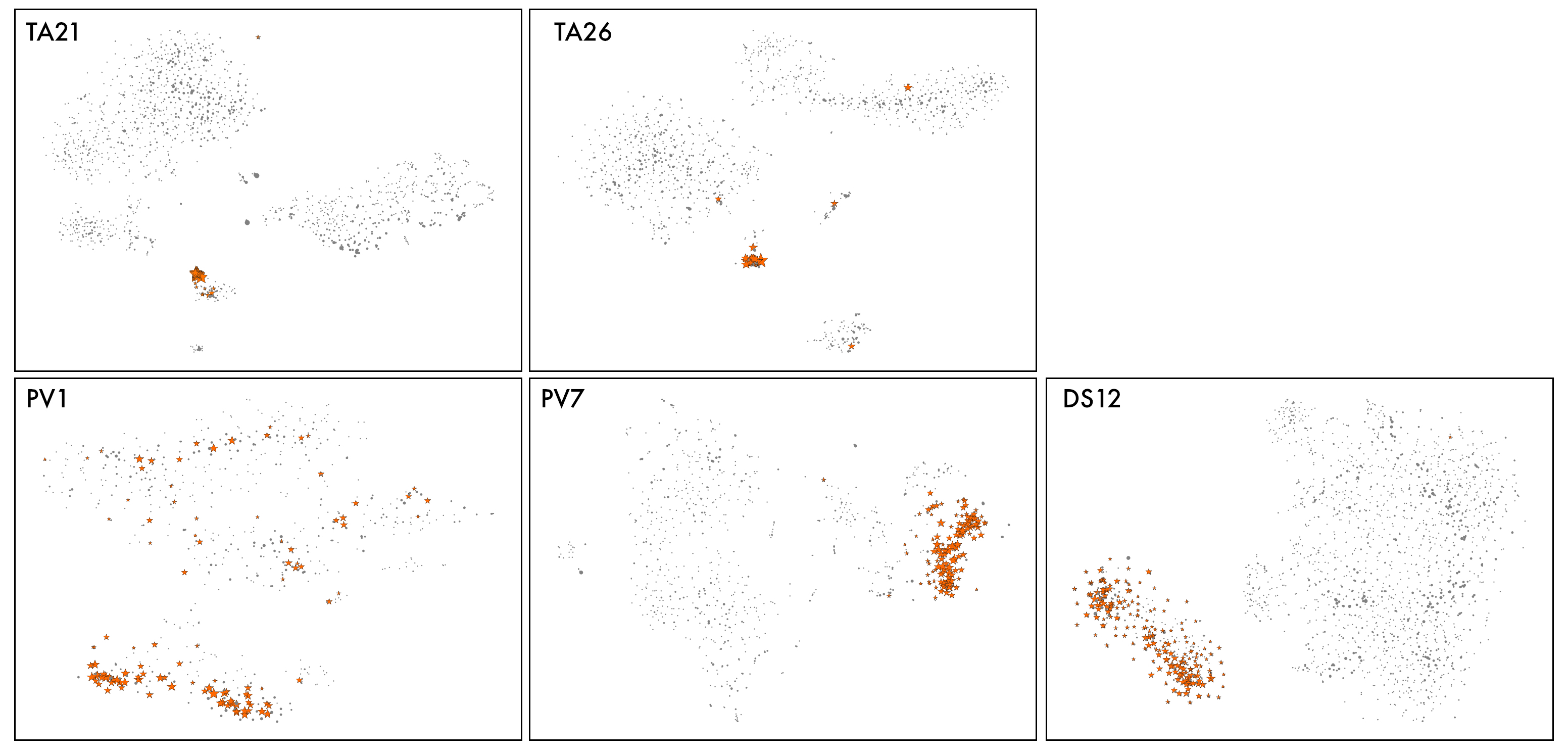

Supplement: FIG S1 [file msphere.00021-22-sf001.jpg]
