# Supplementary material for: Simultaneous Single-Cell Genome and Transcriptome Sequencing of Termite Hindgut Protists Reveals Metabolic and Evolutionary Traits of Their Endosymbionts
Source: mSphere. 2022 Feb 2;7(1):e00021-22. doi: 10.1128/msphere.00021-22 (PMC8809381; doi:10.1128/msphere.00021-22)
Supplement: Supplementary file 3 [file msphere.00021-22-st001.docx]

	TA21	TA26	PV1	PV7	DS12
TotalReads	22.5M	17.7M	25.5M	15.0M	23.3M
#ReadsMapped	530	287	37	60	30

				Reads Mapped #						Reads Mapped %				
ASV	ASV_Taxonomy	Kingdom		TA21	TA26	PV1	PV7	DS12		TA21	TA26	PV1	PV7	DS12
18S_rRNA::TA21	Trichonympha_TA21_sc229	Euk		359	0	0	0	2		67.7%	0.0%	0.0%	0.0%	6.7%
KC494354.1_Tri	Trichonympha	Euk		1	122	0	0	0		0.2%	42.5%	0.0%	0.0%	0.0%
KC494348.1.150	Trichonympha burlesquei 2	Euk		6	6	0	0	0		1.1%	2.1%	0.0%	0.0%	0.0%
KC494352.1.159	Trichonympha sp. 2 ERJ-20	Euk		1	4	0	0	0		0.2%	1.4%	0.0%	0.0%	0.0%
18S_rRNA::PV1	Pyrsonympha_PV1_sc183	Euk		0	0	8	0	0		0.0%	0.0%	21.6%	0.0%	0.0%
Elusimicrobia_	Elusimicrobia_ASV020	Prok		143	0	0	1	0		27.0%	0.0%	0.0%	1.7%	0.0%
Elusimicrobia_	Elusimicrobia_ASV019	Prok		0	96	0	0	0		0.0%	33.4%	0.0%	0.0%	0.0%
Elusimicrobia_	Elusimicrobia_ASV024	Prok		0	0	25	22	0		0.0%	0.0%	67.6%	36.7%	0.0%
Elusimicrobia_	Elusimicrobia_ASV022	Prok		0	0	0	1	0		0.0%	0.0%	0.0%	1.7%	0.0%
Elusimicrobia_	Elusimicrobia_ASV023	Prok		0	0	0	0	5		0.0%	0.0%	0.0%	0.0%	16.7%
Spirochaetes_A	Spirochaetes_ASV093	Prok		10	45	0	0	0		1.9%	15.7%	0.0%	0.0%	0.0%
Spirochaetes_A	Spirochaetes_ASV048	Prok		4	11	0	0	0		0.8%	3.8%	0.0%	0.0%	0.0%
Spirochaetes_A	Spirochaetes_ASV044	Prok		4	0	0	0	0		0.8%	0.0%	0.0%	0.0%	0.0%
Spirochaetes_A	Spirochaetes_ASV064	Prok		1	0	0	0	0		0.2%	0.0%	0.0%	0.0%	0.0%
Spirochaetes_A	Spirochaetes_ASV095	Prok		1	0	0	0	0		0.2%	0.0%	0.0%	0.0%	0.0%
Spirochaetes_A	Spirochaetes_ASV049	Prok		0	1	0	0	0		0.0%	0.3%	0.0%	0.0%	0.0%
Spirochaetes_A	Spirochaetes_ASV042	Prok		0	1	0	0	0		0.0%	0.3%	0.0%	0.0%	0.0%
Spirochaetes_A	Spirochaetes_ASV043	Prok		0	1	0	0	0		0.0%	0.3%	0.0%	0.0%	0.0%
Spirochaetes_A	Spirochaetes_ASV087	Prok		0	0	0	0	4		0.0%	0.0%	0.0%	0.0%	13.3%
Spirochaetes_A	Spirochaetes_ASV082	Prok		0	0	0	0	3		0.0%	0.0%	0.0%	0.0%	10.0%
Spirochaetes_A	Spirochaetes_ASV083	Prok		0	0	0	0	3		0.0%	0.0%	0.0%	0.0%	10.0%
Spirochaetes_A	Spirochaetes_ASV098	Prok		0	0	0	0	3		0.0%	0.0%	0.0%	0.0%	10.0%
Spirochaetes_A	Spirochaetes_ASV071	Prok		0	0	0	0	2		0.0%	0.0%	0.0%	0.0%	6.7%
Spirochaetes_A	Spirochaetes_ASV058	Prok		0	0	0	0	1		0.0%	0.0%	0.0%	0.0%	3.3%
Spirochaetes_A	Spirochaetes_ASV066	Prok		0	0	0	0	1		0.0%	0.0%	0.0%	0.0%	3.3%
Spirochaetes_A	Spirochaetes_ASV070	Prok		0	0	0	0	1		0.0%	0.0%	0.0%	0.0%	3.3%
Spirochaetes_A	Spirochaetes_ASV074	Prok		0	0	0	0	1		0.0%	0.0%	0.0%	0.0%	3.3%
Spirochaetes_A	Spirochaetes_ASV094	Prok		0	0	0	0	1		0.0%	0.0%	0.0%	0.0%	3.3%
Spirochaetes_A	Spirochaetes_ASV096	Prok		0	0	0	0	1		0.0%	0.0%	0.0%	0.0%	3.3%
Spirochaetes_A	Spirochaetes_ASV035	Prok		0	0	0	0	1		0.0%	0.0%	0.0%	0.0%	3.3%
Spirochaetes_A	Spirochaetes_ASV036	Prok		0	0	0	0	1		0.0%	0.0%	0.0%	0.0%	3.3%
Bacteroidetes_	Bacteroidetes_ASV009	Prok		0	0	0	27	0		0.0%	0.0%	0.0%	45.0%	0.0%
Bacteroidetes_	Bacteroidetes_ASV006	Prok		0	0	0	2	0		0.0%	0.0%	0.0%	3.3%	0.0%
Bacteroidetes_	Bacteroidetes_ASV011	Prok		0	0	0	1	0		0.0%	0.0%	0.0%	1.7%	0.0%
16S_rRNA::PV1	Firmicutes_Tyzzerella_PV1	Prok		0	0	4	3	0		0.0%	0.0%	10.8%	5.0%	0.0%
16S_rRNA::PV7	Williamwhitmaniaceae_PV7_	Prok		0	0	0	1	0		0.0%	0.0%	0.0%	1.7%	0.0%
16S_rRNA::PV7	Planctomycete_PV7_sc189	Prok		0	0	0	2	0		0.0%	0.0%	0.0%	3.3%	0.0%
